# Non–beta blocker enantiomers of propranolol and atenolol inhibit vasculogenesis in infantile hemangioma

**DOI:** 10.1172/JCI151109

**Published:** 2022-02-01

**Authors:** Caroline T. Seebauer, Matthew S. Graus, Lan Huang, Alex McCann, Jill Wylie-Sears, Frank Fontaine, Tara Karnezis, David Zurakowski, Steven J. Staffa, Frédéric Meunier, John B. Mulliken, Joyce Bischoff, Mathias Francois

**Affiliations:** 1 Vascular Biology Program, Department of Surgery, Boston Children’s Hospital, Harvard Medical School, Boston, USA.; 2 The David Richmond Laboratory for Cardiovascular Development: Gene Regulation and Editing, The Centenary Institute, The University of Sydney, Camperdown, New South Wales, Australia.; 3 Institute for Molecular Bioscience, Queensland Brain Institute, The University of Queensland, St. Lucia, Queensland, Australia.; 4 Gertrude Biomedical Pty Ltd., Bio21, Melbourne, Victoria, Australia.; 5 Department of Anesthesiology, Critical Care and Pain Medicine Research, Boston Children’s Hospital, Harvard Medical School, Boston, USA.; 6 Department of Plastic and Oral Surgery, Boston Children’s Hospital, Harvard Medical School, Boston, Massachusetts, USA.

**Keywords:** Angiogenesis, Vascular Biology, Drug therapy, Endothelial cells, Transcription

## Abstract

Propranolol and atenolol, current therapies for problematic infantile hemangioma (IH), are composed of R(+) and S(–) enantiomers: the R(+) enantiomer is largely devoid of beta blocker activity. We investigated the effect of R(+) enantiomers of propranolol and atenolol on the formation of IH-like blood vessels from hemangioma stem cells (HemSCs) in a murine xenograft model. Both R(+) enantiomers inhibited HemSC vessel formation in vivo. In vitro, similar to R(+) propranolol, both atenolol and its R(+) enantiomer inhibited HemSC to endothelial cell differentiation. As our previous work implicated the transcription factor sex-determining region Y (SRY) box transcription factor 18 (SOX18) in propranolol-mediated inhibition of HemSC to endothelial differentiation, we tested in parallel a known SOX18 small-molecule inhibitor (Sm4) and show that this compound inhibited HemSC vessel formation in vivo with efficacy similar to that seen with the R(+) enantiomers. We next examined how R(+) propranolol alters SOX18 transcriptional activity. Using a suite of biochemical, biophysical, and quantitative molecular imaging assays, we show that R(+) propranolol directly interfered with SOX18 target gene *trans*-activation, disrupted SOX18-chromatin binding dynamics, and reduced SOX18 dimer formation. We propose that the R(+) enantiomers of widely used beta blockers could be repurposed to increase the efficiency of current IH treatment and lower adverse associated side effects.

## Graphical abstract

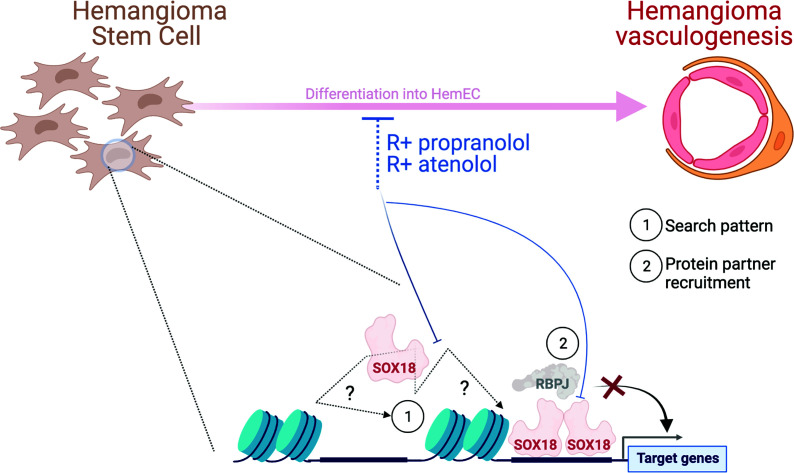

## Introduction

Infantile hemangioma (IH) is a common benign vascular tumor of infancy that occurs in 4%–5% of mature neonates, with a predominance in females and infants of European descent (1). The risk for IH increases with low birth weight and decreasing gestational age (2). Additional risk factors are family history, intrauterine complications, and placental anomalies (3). IHs have a unique life cycle. Lesions are not present at birth. They arise in early infancy (at 3–7 weeks of age), proliferate up to 12 months of age (4), and involute spontaneously. The involuting phase can last several years; in approximately 50%–70% of tumors, there are residua (telangiectasia, fibrofatty tissue, redundant skin, anetoderma, dyspigmentation, or scarring; refs. 5, 6).

In 10%–15% of cases, IHs cause complications such as airway or visual obstruction, cardiac failure, feeding difficulties, ulceration, and disfigurement (1). Propranolol, a nonselective β-adrenergic receptor antagonist, is the first-line treatment for complicated IHs (5). Since its first use for IH in 2008, propranolol has been considered to be more effective and safer than previous medications such as corticosteroids, IFN, or vincristine (7–11). The response rate to propranolol is 96%– 98%. Oral propranolol at 2–3 mg/kg per day after a mean of 6 months of therapy shows complete or nearly complete regression in 60% of cases (1, 7, 12, 13). Recurrence is observed in 10%–15% of cases after discontinuation of therapy (14, 15). Concerning side effects of propranolol are due to its antagonistic effect on β-adrenergic receptors and include sleep disorder, bronchospasm, bradycardia, hypotension, and hypoglycemia. Atenolol, a hydrophilic β1-adrenergic receptor blocker, is used to reduce the potential risk of β2-adrenergic receptor–related side effects (16). There are few studies on the long-term effects of atenolol, and the mechanism of action of atenolol in IH treatment has not to our knowledge been explored.

Propranolol and atenolol are composed of a 1:1 racemic mixture of R(+) and S(–) enantiomers. S(–) enantiomers are potent β-adrenergic receptor antagonists, whereas R(+) enantiomers are largely devoid of beta blocker activity (17–21), with selectivity maintained at 10 μM (22). The mechanism of action of propranolol in IH treatment is incompletely understood. Overman et al. demonstrated that propranolol can act independently of its effect on β-adrenergic receptors by disrupting dimer formation of the transcription factor SRY (sex-determining region Y) box transcription factor 18 (SOX18) (23), which plays an important role in endothelial cell (EC) differentiation during blood vessel development (24, 25) as well as tumor-induced angiogenesis (26). These findings showed that the R(+) enantiomer of propranolol is sufficient to inhibit the differentiation of hemangioma stem cells (HemSCs) to hemangioma ECs (HemECs) in vitro (23). Further, this work also established a SOX18-dependent mechanism in vivo by demonstrating that R(+) propranolol rescues a corneal neovascularization phenotype caused by the altered SOX18 function in Ragged Opossum mutant mice.

To decipher the molecular and cellular mechanism of the non–beta blocker enantiomers in IH, we investigated whether R(+) propranolol and R(+) atenolol inhibit IH vessel formation in vivo and HemSC differentiation in vitro. We also studied the effects of R(+) enantiomers on SOX18 molecular mechanisms in an effort to establish a mode of action and on-target engagement for these compounds on this new molecular target.

## Results

### R(+) propranolol inhibits vasculogenesis in a murine model for IH.

**Figure 1:**
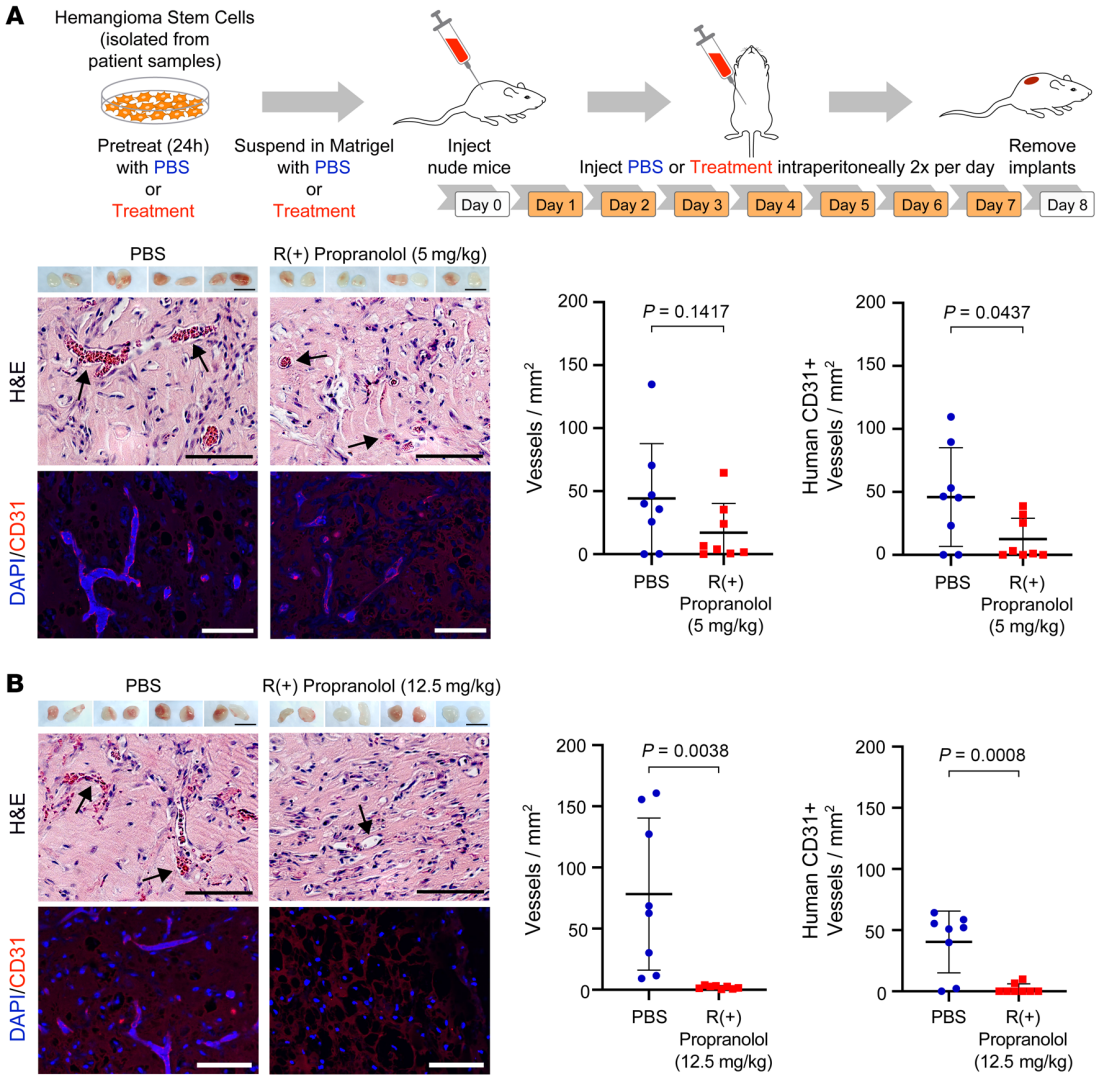
R(+) propranolol inhibits vessel formation in a murine model for IH. (**A**) HemSCs were pretreated with PBS or 10 μM R(+) propranolol for 24 hours, suspended in Matrigel with PBS or 5 μM R(+) propranolol, and then injected into nude mice, with 2 implants/mouse (*n =* 8 mice). The mice were treated with 5 mg/kg R(+) propranolol or an equivalent volume of PBS twice a day as depicted in the schematic. Matrigel implants harvested after 7 days are shown in the top panel of the images. Scale bars: 10 mm. H&E staining indicated fewer blood vessels in the implants of R(+) propranolol–treated mice compared with implants in the control mice (middle panels). Scale bars: 100 μm. Anti–human CD31 staining (red) confirmed the reduced vessel density in R(+) propranolol–treated mice compared with vessel density in control mice (bottom panels). Nuclei were counterstained with DAPI (blue). Scale bars: 100 μm. Graphs show quantification of vessels/mm^2^ in the H&E-stained sections (left) and human CD31^+^ vessels/mm^2^ (right). (**B**) HemSCs were treated as described in **A**. Mice were treated with 12.5 mg/kg R(+) propranolol or the equivalent volume of PBS twice a day. Matrigel implants harvested after 7 days are displayed in the top panel of the images, with 2 implants/mouse (*n =* 8 mice). Scale bars: 10 mm. H&E staining (middle panels) and anti–human CD31 staining (red; bottom panels) showed a significant reduction in vessel density in the implants of R(+) propranolol–treated mice compared with control mice. Scale bars: 100 μm. *P* values were calculated using a 2-tailed, unpaired Student’s *t* test. Data show the mean ± SD. Data were collected for 2 implants in each of 4 mice, leading to an observation sample size of 8 per group.

The potential of the R(+) enantiomer of propranolol to inhibit HemSC to HemEC differentiation in vitro (23), independent of β-adrenergic activity, prompted us to study its efficacy in vivo in an established murine model for IH (see schematic in Figure 1A and refs. 27, 28). HemSCs (isolated from deidentified IH specimen 150A) pretreated with PBS as a control or 10 μM R(+) propranolol for 24 hours were suspended in Matrigel. The concentration was based on amounts for in vitro effects on β-adrenergic receptors. We injected cell/Matrigel suspensions s.c. into the backs of nude mice (2 implants per mouse), as described previously (29, 30). We collected data for 2 implants in each of 4 mice, leading to a observation sample size of 8 per group. After 7 days of twice-daily treatment with 5 mg/kg R(+) propranolol or an equal volume of PBS as a control, the implants were removed, photographed, and sectioned for histology and immunofluorescence (IF) staining. Both groups of implants showed vascularization (Figure 1A, upper panel), but counting red blood cell–filled lumens in H&E-stained sections revealed a trend toward fewer vessels in the implants from mice treated with 5 mg/kg R(+) propranolol (Figure 1A, middle panel). To distinguish between vessels formed by the implanted human HemSCs and vessels derived from the host (mouse), we stained sections with anti–human CD31, an antibody specific for human ECs that has no detectable cross-reactivity with murine ECs (31). This method revealed vessels lined with human ECs and showed a reduced number of human vessels in the implants of the mice treated with R(+) propranolol (Figure 1A, lower panel). We increased the dosage of R(+) propranolol in the next experiment to more closely approximate the dosage used in patients with IH (32). Mice were treated twice a day with 12.5 mg/kg R(+) propranolol or an equal volume of PBS as a control. After 7 days, implants from mice treated with 12.5 mg/kg R(+) propranolol showed almost no vascularization macroscopically, whereas the implants of the PBS-treated control mice were vascularized (Figure 1B images, upper panel). Counting red blood cell–filled lumens in H&E-stained sections (Figure 1B images, middle panel), as well as anti–human CD31^+^ vessels (Figure 1B images, lower panel), revealed a significant reduction of total vessel density and human vessel density in the implants from mice treated with 12.5 mg/kg R(+) propranolol. R(+) propranolol had no effect on the number of mouse CD31^+^ vessels detected in the Matrigel implants (Supplemental Figure 1A; supplemental material available online with this article; https://doi.org/10.1172/JCI151109DS1). Murine vessels were probably recruited into implants by the high level of VEGF-A secreted by HemSCs (33). Propranolol, atenolol, and their respective R(+) enantiomers had no effect on the levels of VEGF-A secreted by HemSC 150A (Supplemental Figure 1B), providing a potential explanation for the uninhibited recruitment of murine vessels. Thus, R(+) propranolol inhibited the formation of perfused human CD31^+^ vessels from HemSCs. The effect was dose dependent and likely independent of β-adrenergic effects, since the R(+) enantiomer is a poor antagonist (20).

### R(+) propranolol does not affect hemangioma-derived pericyte differentiation.

**Figure 2:**
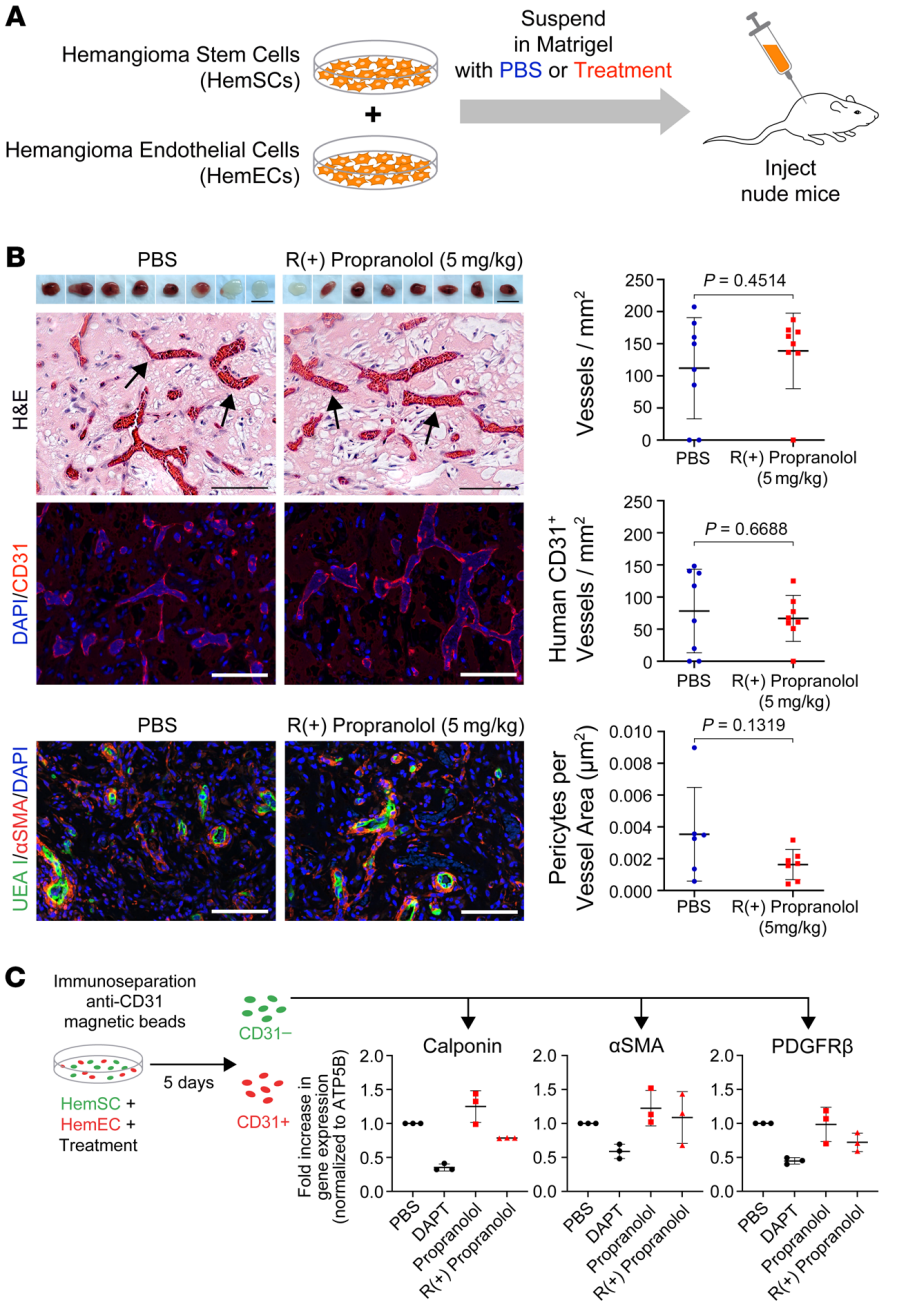
R(+) propranolol does not affect HemSC to HemPericyte differentiation. (**A**) HemSCs and HemECs (1:1) were suspended in Matrigel and injected into nude mice, with 2 implants/mouse (*n =* 8 mice). The mice were treated with 5 mg/kg R(+) propranolol or an equivalent volume of PBS twice a day. Matrigel implants harvested after 7 days are displayed in the top panel of the images. Scale bars: 10 mm. H&E staining showed similar vessel density in the implants of R(+) propranolol–treated mice compared with vessel density in the implants of control mice (middle panels). Scale bars: 100 μm. Anti–human CD31 staining (red) confirmed the similar number of blood vessels in R(+) propranolol–treated mice and control mice (bottom panels). Nuclei were counterstained with DAPI (blue). Scale bars: 100 μm. *P* values were calculated using a 2-tailed, unpaired Student’s *t* test. Data were collected for 2 implants in each of 4 mice, leading to an observation sample size of 8 per group. (**B**) Implant sections stained with UEA I (green) and anti-αSMA (red) showed similar pericyte coverage per vessel area in mice treated with PBS (*n =* 7 mice) or R(+) propranolol (*n =* 6 mice). Nuclei were counterstained with DAPI (blue). Scale bars: 100 μm. *P* values were calculated by 2-tailed, unpaired Student’s *t* test. Only implants showing vessel formation were used for further analysis [*n =* 7 PBS implants; *n =* 6 R(+) propranolol implants]. Graphs show quantification of vessels/mm^2^ in the H&E-stained sections (top), human CD31^+^ vessels/mm^2^ (middle), and pericytes/vessel area (bottom). (**C**) qPCR showed that treatment with propranolol or its R(+) enantiomers (10 μM) did not affect the expression of pericyte markers (calponin, PDGFRβ, and αSMA) in HemSCs cocultured with HemECs. Coculturing was conducted for 5 days: CD31^+^ cells were removed by magnetic beads before RNA extraction of the CD31^–^ cells as shown in the schematic. DAPT (10 μM) served as a positive control. Data from 3 independent experiments were plotted. Statistical significance was determined by 1-way ANOVA with Dunnett’s multiple-comparison test. *P* values can be found in Supplemental Figure 2C. Data in all graphs show the mean ± SD.

We investigated whether R(+) propranolol inhibits HemSC to hemangioma-derived pericyte (HemPericyte) differentiation. Boscolo et al. demonstrated that HemSCs in contact with ECs differentiate into IH pericyte–like cells via NOTCH signaling (28, 34). We surmised that providing HemSCs direct contact with HemECs in the Matrigel suspension would bypass the need for EC differentiation; hence, vessel formation would rely solely on HemSC to HemPericyte differentiation. To test this supposition, HemSCs (150A) and HemECs (150A) at a 1:1 ratio were suspended in Matrigel and injected into nude mice (*n =* 4 mice/group), 2 implants/mouse (see schematic in Figure 2A). The mice were treated with 5 mg/kg R(+) propranolol or an equal volume of PBS twice a day. After 7 days, the Matrigel implants were harvested, photographed, and sectioned for histology and IF staining. Both groups of implants showed robust vascularization (Figure 2A images, upper panel). Counting of red blood cell–filled lumens in H&E-stained sections (Figure 2A images, middle panel) and staining with anti–human CD31 (Figure 2A images, lower panel) demonstrated no difference in vessel density in the implants of mice treated with 5 mg/kg R(+) propranolol compared with those treated with the PBS control. To determine whether the vessels differed in mural cell coverage, sections from the implants [*n =* 7 PBS-treated; *n =* 6 R(+) propranolol-treated] were costained with UEA1 (a plant lectin that binds avidly to human ECs but not to murine ECs) and the pericyte/smooth muscle marker α smooth muscle actin (αSMA) (Figure 2B). We calculated the vessel area and determined the number of perivascular cells per vessel area in μm^2^. Matrigel implants of mice treated with 5 mg/kg R(+) propranolol showed perivascular cell coverage similar to that observed in the implants of the PBS-treated control mice.

To identify whether propranolol or its R(+) enantiomer affected HemSC to HemPericyte differentiation in vitro, we cocultured HemSCs (150A) with HemECs (150A) in the presence or absence of drug. After 5 days, we removed the HemECs by anti-CD31 dynabeads (see schematic in Figure 2C). RNA isolated from the CD31^–^ cells was used to detect the IH pericytic markers calponin, PDGFRβ, and αSMA by quantitative reverse transcriptase PCR (qPCR) (35). The qPCR analysis of 3 biological replicates showed no significant difference in the expression of the pericyte markers in cocultured cells treated with propranolol or R(+) propranolol compared with the PBS control (Figure 2C; for *P* values, see Supplemental Figure 2C). Treatment with DAPT, a γ-secretase inhibitor that blocks NOTCH signaling, reduced the expression of pericyte markers as expected (28). In summary, these data confirm that HemSCs cocultured in direct contact with ECs expressed pericyte markers but that propranolol and its R(+) enantiomer had no effect on HemSC to HemPericyte differentiation in vitro or on HemSC plus HemEC vessel formation in vivo.

To address, whether R(+) propranolol inhibits the angiogenic activities of differentiated HemECs, we tested the effect of R(+) propranolol on HemEC proliferation and tube formation. Previous work showed that racemic propranolol (10 μM) inhibited HemEC proliferation but had no effect on the proliferation of HemSCs (36). R(+) propranolol reduced the proliferation of HemECs to the same extent as racemic propranolol (Supplemental Figure 2A). Additionally, R(+) propranolol inhibited HemEC tube formation (Supplemental Figure 2B ).

### R(+) atenolol inhibits HemEC differentiation in vitro and vessel formation in vivo.

**Figure 3:**
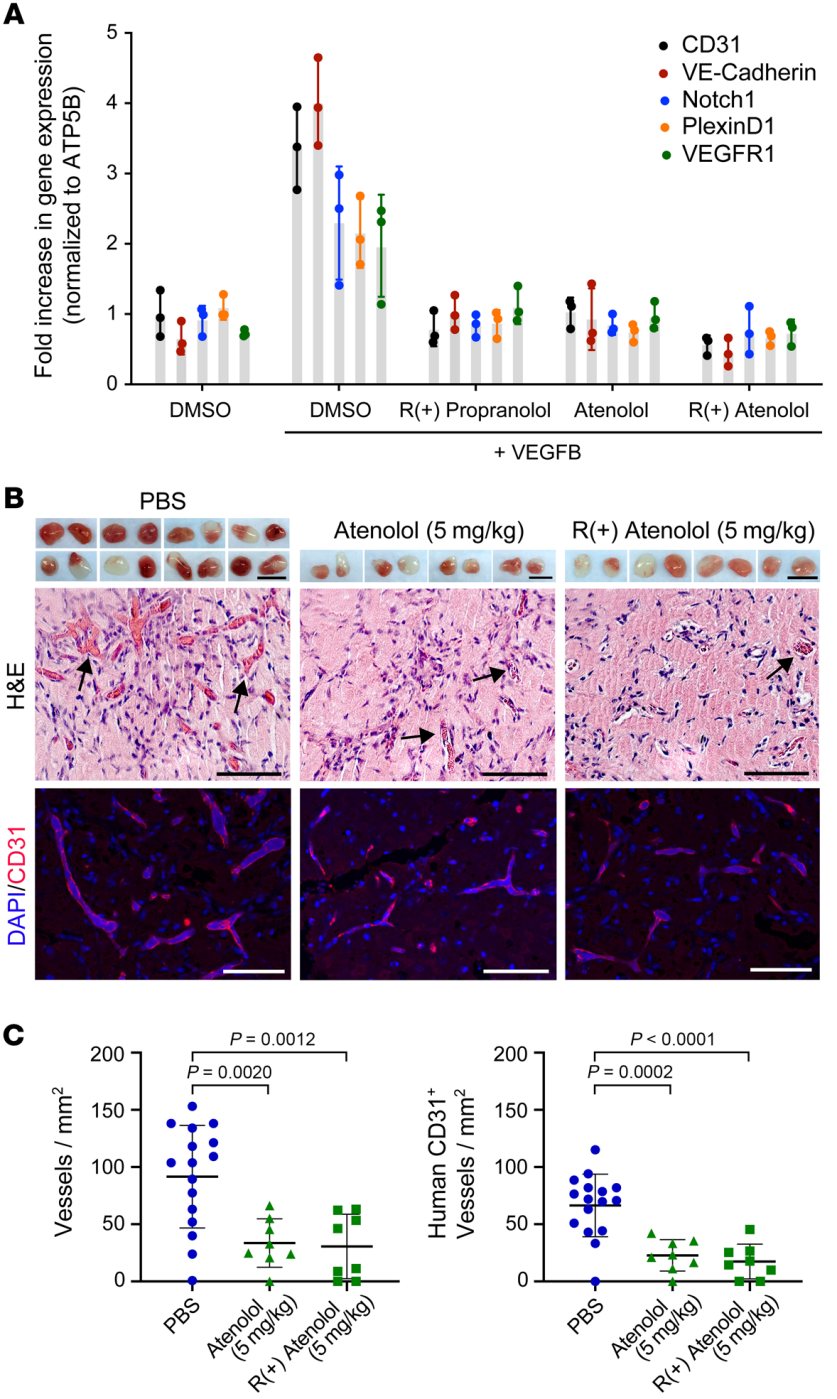
R(+) atenolol inhibits hemangioma endothelial differentiation in vitro and vessel formation in vivo. (**A**) Atenolol and its purified R(+) enantiomer, both tested at 5 μM, inhibited endothelial differentiation of HemSCs isolated from 2 IH tumor specimens as effectively as did R(+) propranolol. R(+) propranolol served as a positive control for inhibition. The endothelial differentiation markers CD31 and VE-cadherin and the hemangioma endothelial markers NOTCH1, PlexinD1, and VEGFR1 under each treatment condition in 3 biological replicates, determined by qPCR, were standardized as previously described (76). The HemSC-to-endothelial differentiation assay was conducted 2 separate times with HemSC 167 and once with HemSC 165, providing 3 data points. Statistical significance was determined by 1-way ANOVA with Bonferroni’s post hoc test. *P* values are listed in Supplemental Figure 3. (**B**) HemSCs were pretreated with PBS, 10 μM atenolol, or 10 μM R(+) atenolol 24 hours before the experiment and were then suspended in Matrigel with PBS, 5 μM atenolol, or 5 μM R(+) atenolol and injected into nude mice, with 2 implants per mouse (see schematic in Figure 1A; *n =* 16 PBS-treated HemSCs, *n =* 8 atenolol-treated HemSCs, *n =* 8 R(+) atenolol–treated HemSCs). The mice were treated with 5 mg/kg atenolol, 5 mg/kg R(+) atenolol, or an equal volume of PBS twice a day. Matrigel implants harvested after 7 days are shown in the top panels of the images. Scale bars: 10 mm. Images also show H&E staining (middle panels) and anti–human CD31 staining (red, bottom panels), with nuclei counterstained with DAPI (blue). Scale bars: 100 μm. Data were collected for 2 implants in each of 4 mice, leading to an observation sample size of 8 per treatment group and 16 in the control group. (**C**) Quantification of vessel density based on H&E staining (middle panels) and anti–human CD31 staining (bottom panels) showed a significant reduction in vessel density in the implants of atenolol- and R(+) atenolol–treated mice versus implants of control mice. Statistical analysis was performed using 1-way ANOVA with Dunnett’s multiple-comparison test.

Atenolol, a selective β1-adrenergic receptor antagonist, offers advantages over the nonselective adrenergic blocker propranolol in the treatment of IH, because it does not cause β2-related side effects such as hypoglycemia and bronchospasm. Like propranolol, atenolol exists in a racemic mixture of R(+) and S(–) enantiomers; the R(+) enantiomer is mostly devoid of beta blocker activity (21). Therefore, we investigated whether the mechanism of action proposed for propranolol also occurs with atenolol (23). To determine whether atenolol inhibits hemangioma endothelial differentiation independently of its beta blocker effects, we assessed VEGF-B–induced HemSC to endothelial differentiation using HemSCs isolated from 2 IH specimens (specimens 165, 167) in the presence of atenolol, its R(+) enantiomer, and R(+) propranolol as a positive control. Atenolol and its R(+) enantiomer recapitulated the effect of R(+) propranolol on the endothelial differentiation of HemSCs (Figure 3A). Both inhibited the expression of the endothelial differentiation markers CD31, VE-cadherin, and the expression of the hemangioma endothelial markers NOTCH1, VEGFR1, and plexin D1 compared with the DMSO control (for *P* values, see Supplemental Figure 3).

To extend these findings, we tested the effect of atenolol and R(+) atenolol in the murine model described in 2.1. HemSCs (150A), pretreated with PBS as a control, 10 μM atenolol, or 10 μM R(+) atenolol 24 hours before the experiment, were suspended in Matrigel and injected s.c. into the backs of nude mice (*n =* 8 in the control group, *n =* 4 per treatment group), with 2 implants per mouse. After 7 days of twice-daily treatment with 5 mg/kg atenolol, 5 mg/kg R(+) atenolol, or an equal volume of PBS as a control, the implants were removed, photographed, and sectioned for histology and IF staining. Both treatment groups showed lighter-colored implants, whereas the implants in the PBS control group showed distinct vascularization (Figure 3B, upper panels). Our assessment of red blood cell–filled lumens in H&E-stained sections (Figure 3B, middle panels) and anti–human CD31^+^ vessels (Figure 3B, lower panels) revealed a significant reduction in total vessel density and human vessel density in implants from the atenolol-treated mice as well as from the R(+) atenolol–treated mice compared with the PBS-treated mice (Figure 3C). R(+) atenolol treatment had no effect on the number of murine CD31^+^ vessels in the Matrigel implants (Supplemental Figure 1A).

### The orally active SOX18 inhibitor Sm4 suppresses vessel formation in a murine model of IH.

**Figure 4:**
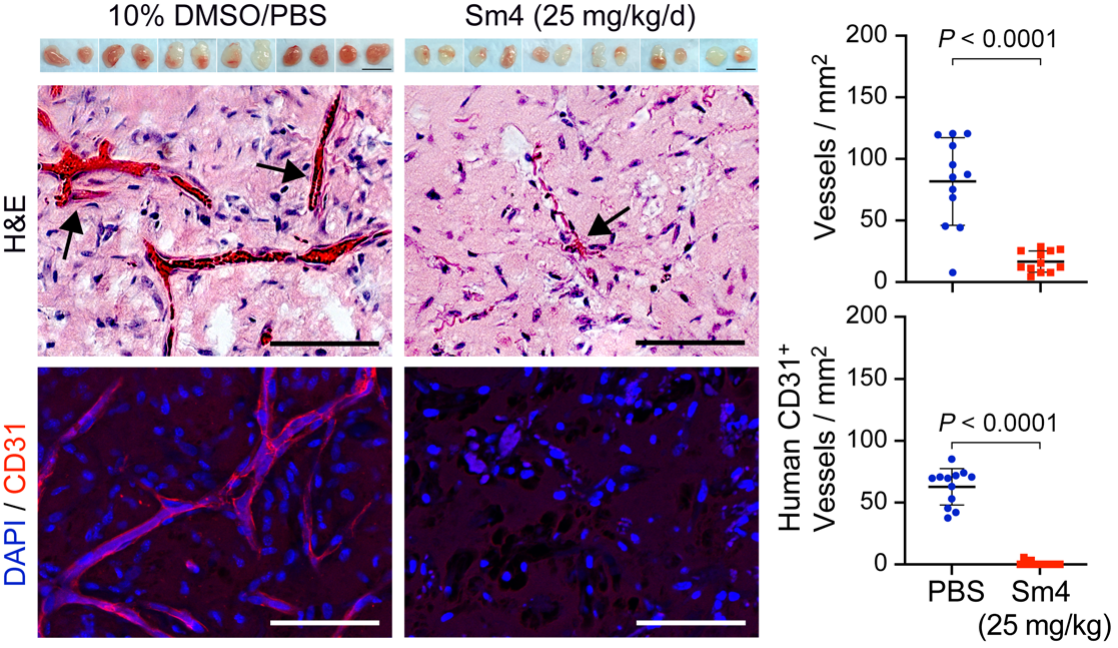
The orally active SOX18 inhibitor Sm4 suppresses vessel formation in a murine model for IH. HemSCs were pretreated with 10% DMSO in PBS or 10 μM Sm4 for 24 hours, suspended in Matrigel with 10% DMSO in PBS or 5 μM Sm4 and injected into nude mice, with 2 implants per mouse (*n =* 12). The mice were treated with 25 mg/kg Sm4 or an equivalent volume of 10% DMSO in PBS once a day by oral gavage. Matrigel implants harvested after 7 days are shown in the top panels. Scale bars: 10 mm. H&E staining (middle panels) and anti–human CD31 staining (red; lower panels) showed a significant reduction in vessel density in the implants from Sm4-treated mice compared with those from control mice. Nuclei were counterstained with DAPI (blue). Scale bars: 100 μm. Graphs show quantification of vessels/mm^2^ in the H&E-stained sections (top) and human CD31^+^ vessels/mm^2^ (bottom). *P* values were calculated by 2-tailed, unpaired Students’ *t* test. Data show the mean ± SD. Data were collected for 2 implants in each of 6 mice, leading to an observation sample size of 12 per group.

To further assess the possibility of adrenergic involvement, we tested in the murine model for IH described in Figure 1A the small molecule Sm4, an orally active SOX18 inhibitor that directly disrupts SOX18 interaction with a subset of binding partners (37). We administered Sm4 by oral gavage at a dose of 25 mg/kg once a day, as this concentration has been used successfully to reduce tumor vascular density in a mouse preclinical model of breast cancer (38). HemSCs (150A), pretreated with 10% DMSO and PBS as a control or 10 μM Sm4 24 hours before the experiment, were suspended in Matrigel and injected s.c. into the backs of nude mice (*n =* 6 per group), with 2 implants per mouse. Data were collected for 2 implants in each of 6 mice, leading to an observation sample size of 12 per group. After 7 days of once-daily treatment with 25 mg/kg Sm4 or an equal volume of 10% DMSO and PBS as a control, the implants were removed, photographed, and sectioned for histology and IF staining. Implants from mice treated with 25 mg/kg/d Sm4 showed almost no vascularization macroscopically, whereas the implants of the PBS-treated control mice were vascularized (Figure 4, upper panel). Counting red blood cell–filled lumens in H&E-stained sections (Figure 4, middle panel), as well as anti–human CD31^+^ vessels (Figure 4, lower panel), revealed a significant reduction in total vessel density and human vessel density in the implants from mice treated with 25 mg/kg Sm4 once a day. We observed no effect of Sm4 treatment on the density of mouse CD31^+^ vessels in the Matrigel implants (Supplemental Figure 4A). We measured body weight and glucose levels daily before oral gavage. Sm4 treatment did not affect body weight or glucose levels when compared with mice treated with 10% DMSO and PBS (Supplemental Figure 4B). These results recapitulate the in vivo effects of 12.5 mg/kg R(+) propranolol and 12.5 mg/kg R(+) atenolol and support a role for SOX18 in IH vasculogenesis.

### R(+) propranolol disrupts SOX18 transcriptional activity via a perturbation of chromatin binding dynamics and protein partner recruitment.

**Figure 5:**
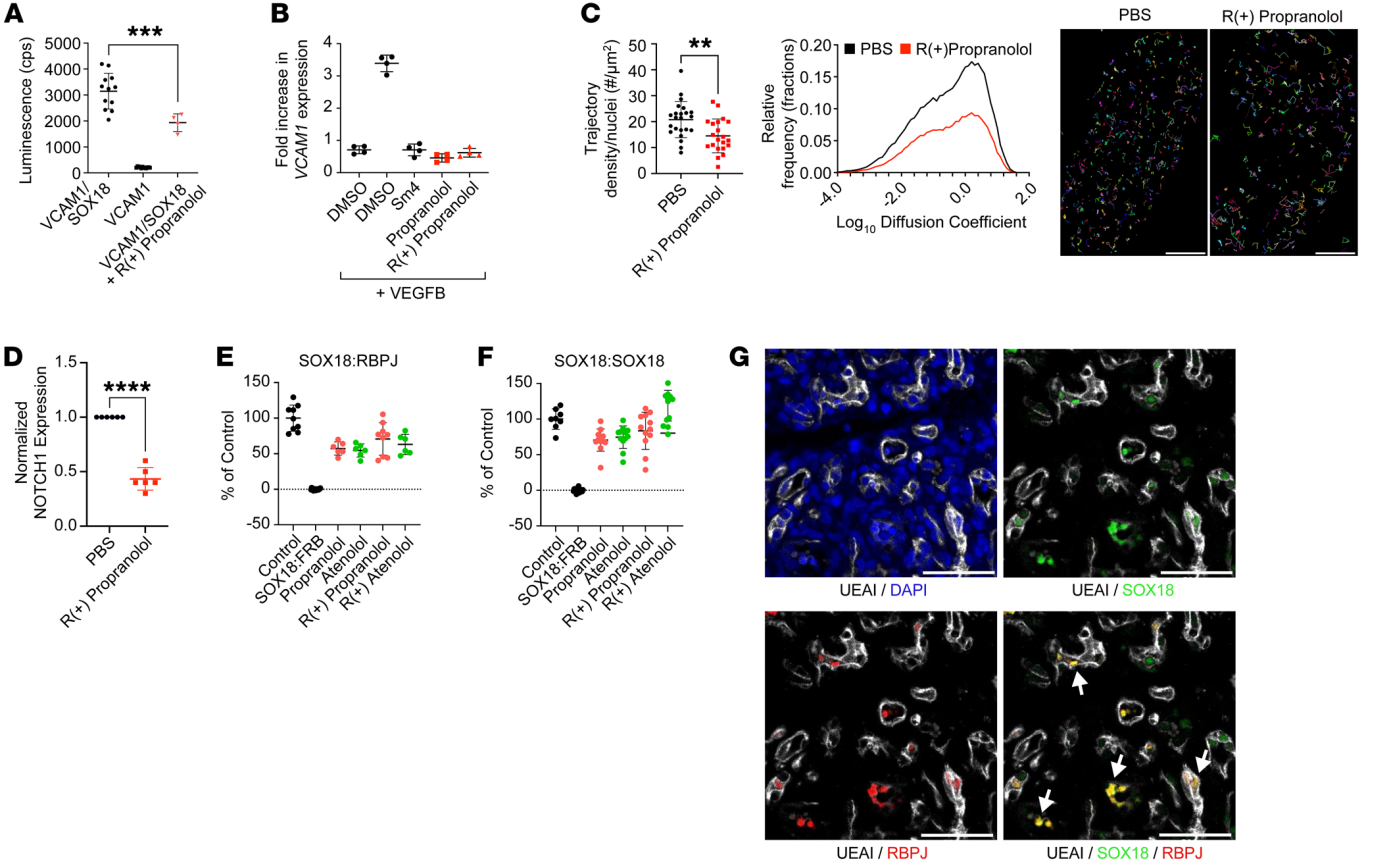
R(+) enantiomers disrupt SOX18 activity. (**A**) SOX18 activated the transcription of VCAM1 in COS-7 cells (luciferase reporter assay); R(+) propranolol (20 μM) inhibited SOX18-driven transcription. The means and SDs are as follows: VCAM1/SOX18, 3148 ± 688; VCAM1, 212 ± 24.8; VCAM1/SOX18 plus R(+) propranolol, 1934 ± 341. Statistical significance was determined by 1-way ANOVA with Tukey’s multiple-comparison test. (**B**) Expression of *VCAM1* was increased by VEGF-B–induced endothelial differentiation of HemSCs from 2 IH tumor specimens. The SOX18 inhibitor Sm4, propranolol, and R(+) propranolol (each tested at 5 μM) reduced *VCAM1* mRNA levels to those of undifferentiated HemSCs. mRNA transcript levels were determined by qPCR and standardized as described previously (76). Statistical significance was determined by 1-way ANOVA with Bonferroni’s post hoc test. *P* values are listed in the table in Supplemental Figure 5E. (**C**) Halo-tagged SOX18 chromatin binding dynamics and diffusion coefficients were measured by SMT in live HeLa cells in the absence or presence of R(+) propranolol. Trajectory density, diffusion coefficient frequency, and individual images show single-molecule tracks that are pseudocolored across the nucleus. Scale bars: 4 μm. **P < 0.005, by Welch’s *t* test on the basis of 4 technical replicates with 6 cells per replicate per condition (*n* ≥20 cells). (**D**) qPCR analysis of NOTCH1 in HemSCs isolated from 6 different IH specimens, differentiated for 8 days with VEGF-B and then treated for 2 hours with or without 20 μM R(+) propranolol. Three technical replicates were performed on the 6 biological replicates. ****P < 0.0001, by paired Student’s *t* test. (**E** and **F**) In the AlphaLISAScreen assay, racemic propranolol, racemic atenolol, and the respective R(+) enantiomers were tested at 20 μM for effects on SOX18:RBPJ (**E**) and SOX18:SOX18 (**F**) PPI. Statistical significance was determined by 1-way ANOVA followed by Dunnett’s post hoc test. *P* values can be found in the table in Supplemental Figure 5F. Data show the mean ± SEM. (**G**) A FFPE tissue section (5 μm) from a patient with IH (female, 5.5 months old, proliferating phase of IH, no propranolol treatment) was stained with anti-SOX18 (1:50, green), anti-RBPJ (1:50, red), and UEA I (1:50, white; to stain human ECs). DAPI (blue) was used to visualize nuclei. Yellow arrows point to double-positive cells (SOX18^+^RBPJ^+^). Scale bars: 50 μm.

To assess the effect of R(+) enantiomers on SOX18 transcriptional activity, we used an in vitro luciferase reporter gene fused to a synthetic human Vcam1 promoter fragment to provide a readout for SOX18 transcriptional activity (Figure 5 and ref. 39). We tested the effect of R(+) propranolol on the SOX18-dependent activation of Vcam1 promoter–driven luciferase activity, which decreased the SOX18-dependent transactivation of Vcam1 promoter activity by approximately 50% (Figure 5A**.**). Consistent with this result, propranolol and its R(+) enantiomer inhibited the endogenous mRNA expression of *VCAM1* in the HemSC-to-EC differentiation assay (Figure 5B; for *P* values, see Supplemental Figure 5E). Sm4, a SOX18 inhibitor, served as a positive control (37, 38). The results from these assays showed that R(+) enantiomer of propranolol interfered with the transactivation of the SOX18 direct target gene *VCAM1*.

In order to establish the proof of concept that R(+) propranolol directly engages with the SOX18 protein, we took advantage of a single-molecule tracking (SMT) assay (40, 41). This method uses highly inclined and laminated optical sheet (HILO) microscopy to assess the trajectory and dwell times of individual transcription factors on the chromatin at single-molecule resolution in real time in a living cell (40). Briefly, we transfected HeLa cells with a SOX18-HaloTag reporter protein to assess SOX18 chromatin binding dynamics and diffusion profiles. Two types of imaging acquisition were performed: (a) fast tracking (20 ms), which enabled us to identify free and bound fractions of SOX18 protein, and (b) slow tracking (500 ms), which enabled us to define 2 distinct types of behaviors within the bound population on the chromatin. These are referred to as either long dwell times, meaning specific binding, or short dwell times, meaning nonspecific binding. Long dwell times previously reported for SOX2 (40) typically range from 5 to 12 seconds and have been described as corresponding to the process of transcriptional regulation per se. By contrast, short dwell times (<1 second) relate to the gene search mechanism on the chromatin, whereby SOX2 surveys the genome to identify target genes. SOX18 chromatin binding dynamics has been characterized in depth using an imaging pipeline that takes advantage of SMT, number and brightness, and cross-correlation raster imaging spectroscopy (41). Here, we focused on the effect that R(+) propranolol has on SOX18 molecular behavior. We found that R(+) propranolol dramatically reduced the density of SOX18 trajectories in the cell nuclei observed (Figure 5C, left). This indicates that the compound engaged with SOX18 in living cells, impeding the transcription factor’s ability to survey the chromatin efficiently because of a lowered density of molecules within the bound fraction. In contrast, R(+) propranolol had no significant effect on SOX18 long- or short-lived dwell times or on the ratio of long-lived/short-lived dwell times (Supplemental Figure 5B). Further, after quantifying the diffusion coefficient of SOX18 (Figure 5C, right graph, and Supplemental Figure 5C), we observed a decrease in the number of trajectories in cells treated with R(+) propranolol (Figure 5C, right graph, PBS: 93,679; R(+) propranolol: 50,920; and Supplemental Figure 5A). Interestingly, R(+) propranolol did not give rise to a major shift in the ratio of bound to free populations (Supplemental Figure 5D), suggesting that this compound affects both the bound and the unbound fraction.

This observation of the interference in SOX18 chromatin-binding dynamics was paralleled by a marked transcriptional repression of its direct target gene NOTCH1. Following 2 hours of R(+) propranolol treatment (20 μM) of HemSCs on day 8 of differentiation, the level of NOTCH1 transcripts was reduced by approximately 60% (Figure 5D). Here, we established a temporal relationship, whereby the time necessary to drive changes in SOX18 molecular behavior is also sufficient to cause a perturbation of its transcriptional output. This suggests that compromising the ability of SOX18 to establish functional pools navigating the chromatin environment is an efficient molecular strategy to disrupt its transcriptional activity.

Previous work has reported that multiple disruptions of simultaneous protein-protein interactions (PPIs) with a small compound are an efficient means of inhibiting SOX18 activity. In particular, SOX18 dimer assembly and recruitment of recombinant signal binding protein for immunoglobulin κ J region (RBPJ) are 2 key PPIs that are interfered with by SOX18 small-molecule inhibitors (23, 37, 38). RBPJ is the main effector of NOTCH signaling and has been shown to be a specific SOX18 dimer protein partner (37, 38). The molecular role of the SOX18 dimer is particularly important in vascular development, since it defines a specific endothelial transcriptional signature (42). To gain molecular insights into propranolol’s mode of interference in SOX18, we next used a homogenous assay known as the AlphaLISA Screen to measure pairwise PPIs. We quantified AlphaScreen signal on both SOX18:RBPJ and SOX18:SOX18 protein pairs in the presence of propranolol, atenolol, and each R(+) enantiomer (Figure 5, E and F). In this assay, we used SOX18-FKBP rapamycin-binding protein (FKB) interaction as a negative control, since there is no interaction between these 2 proteins, and 100% of the binding activity (maximum AlphaScreen signal intensity) was defined in the DMSO control condition for either the SOX18 homodimer or the SOX18:RBPJ heterodimer. Racemic propranolol and racemic atenolol tested at 50 μM reduced SOX18:RBPJ dimer formation by 40%–45%; R(+) propranolol and R(+) atenolol showed comparable inhibition (Figure 5E). The same compounds showed 16%–30% inhibition of the SOX18:SOX18 homodimer (Figure 5E; for inhibition percentages and *P* values, see Supplemental Figure 5F). These in vitro results demonstrate that the R(+) enantiomers inhibited SOX18:RBPJ heterodimer formation and had a milder effect on SOX18 homodimer assembly. Altogether, the combination of SMT and protein interaction assays established a firm proof of concept of on-target engagement for R(+) propranolol on its molecular target, the SOX18 transcription factor.

To assess the physiological relevance of RBPJ in the context of hemangioma, we analyzed both RBPJ and SOX18 expression patterns in formalin-fixed, paraffin-embedded (FFPE) sections from patients with IH. Our goals were to: (a) determine whether RBPJ colocalizes with SOX18 in IH vessels and (b) if SOX18^+^RBPJ^+^ cells are affected by propranolol therapy. Indeed, SOX18^+^ (green)/RBPJ^+^ (red) ECs (white) were detected in the proliferating phase of IH by IF (Figure 5G). Matching the treatment group with age at surgery revealed no significant differences in the number of SOX18^+^, RBPJ^+^, or SOX18^+^RBPJ^+^ cells in the age-matched cohort of 13 IH specimens from patients who received no pharmacologic therapy and 13 IH specimens from patients who received propranolol therapy (Supplemental Figure 6). Statistical comparisons of parameters were performed using multivariable median regression, while adjusting for treatment duration and clustering by matching ID in the analysis (43). These results show coexpression SOX18 and RBPJ but do not support an effect of propranolol therapy on the number of SOX18^+^ or RBPJ^+^ cells in IH. Importantly, this observation further validates in patients with IH the existence of a subset of cells in which SOX18:RBPJ is likely to be targeted by small-molecule inhibitors.

### R(+) propranolol and R(+) atenolol inhibit IH vasculogenesis but not body weight or glucose levels.

**Figure 6:**
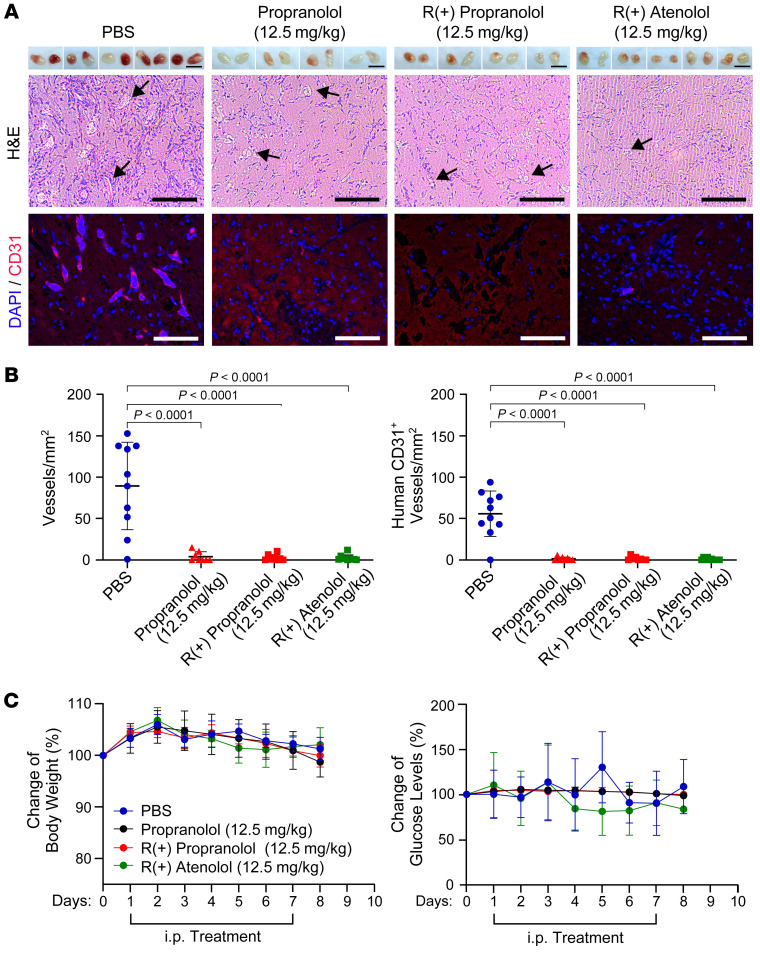
R(+) propranolol and R(+) atenolol inhibit IH vasculogenesis but not body weight or glucose levels. (**A**) HemSCs were pretreated with PBS or 10 μM treatment drug 24 hours before the experiment, suspended in Matrigel with PBS or 5 μM treatment drug, and injected into nude mice, with 2 implants/mouse [*n =* 10 PBS-treated mice, *n =* 8 propranolol-treated mice, *n =* 8 R(+) propranolol–treated mice, *n =* 10 R(+) atenolol–treated mice]. The mice were treated with 12.5 mg/kg propranolol, 12.5 mg/kg R(+) propranolol, 12.5 mg/kg R(+) atenolol, or an equal volume of PBS twice a day. Matrigel implants harvested after 7 days are displayed in the top panels of th images. The PBS control implants in **A** are also shown in Figure 3A, because the 5 mg/kg atenolol group shown in Figure 3B was run at the same time as the groups in **A**. Scale bars: 10 mm. Images show H&E staining (middle panels) and anti–human CD31 staining (red; bottom panels), with nuclei counterstained with DAPI (blue). Scale bars: 100 μm. (**B**) Quantification of vessel density based on H&E staining (**A**, middle panels) and anti–human CD31 staining (**A**, bottom panels) showed that R(+) atenolol was as effective as R(+) propranolol and propranolol in inhibiting vessel formation. Statistical significance was determined by 1-way ANOVA with Dunnett’s multiple-comparison test. *P* values are listed in the table in Supplemental Figure 5E. (**C**) Body weight and glucose levels were measured daily. Neither propranolol, R(+) propranolol, or R(+) atenolol affected body weight or glucose levels of nude mice. Data show the mean ± SD in all graphs. Data were collected for 2 implants in each mouse, leading to an observation sample size of 8 in the propranolol and R(+) propranolol treatment groups and 10 in the atenolol and PBS control groups.

Next, we investigated whether the R(+) enantiomers have an effectiveness similar to that of propranolol at the dosage used in patients with IH and whether they differ in their side effect profiles. Therefore, we used the murine model described in Figure 1A to observe the effects of 12.5 mg/kg propranolol, 12.5 mg/kg R(+) propranolol, and 12.5 mg/kg R(+) atenolol administered twice a day. HemSCs (150A), pretreated with PBS as a control or 10 μM of the assigned treatment for 24 hours, were suspended in Matrigel with the assigned treatment drug or PBS as a control. The cell/Matrigel suspensions were injected s.c. into the backs of nude mice (*n =* 5 PBS control group mice, *n =* 4 propranolol-treated mice, *n =* 4 R(+) propranolol–treated mice, *n =* 5 R(+) atenolol–treated mice), with 2 implants per mouse. After 7 days of the assigned treatment or an equal volume of PBS as a control, the implants were removed, photographed, and sectioned for histology and IF staining. First, we observed a dose-dependent effect for R(+) atenolol: 12.5 mg/kg R(+) atenolol, compared with 5 mg/kg treatment (Figure 3), resulted in Matrigel implants with little to no vascularization macroscopically (Figure 6A, upper panel). Furthermore, quantification of the vessel density in H&E-stained (Figure 6A, middle panel) and anti–human CD31–stained (Figure 6A, lower panel) sections revealed fewer vessels (Figure 6B) than were seen with 5 mg/kg R(+) atenolol treatment (Figure 3B). Second, our findings demonstrate that 12.5 mg/kg R(+) atenolol was similar to 12.5 mg/kg R(+) propranolol and 12.5 mg/kg propranolol in preventing vessel formation in the murine IH model, as observed macroscopically (Figure 6A, upper panel) and confirmed by assessment of red blood cell–filled lumens in the H&E-stained sections (Figure 6A, middle panel) as well as by anti–human CD31 staining (Figure 6B, lower panel). All 3 treatment conditions showed a significant reduction of vessel density compared with that seen in the PBS-treated control mice (Figure 6B). Throughout the experiment, we measured body weight and glucose levels daily before the morning i.p. injections. Immunodeficient mice treated with propranolol, R(+) propranolol, or R(+) atenolol showed no change in body weight or glucose levels compared with the PBS-treated immunodeficient mice (Figure 6C). These findings show that both R(+) propranolol and R(+) atenolol were effective in reducing vasculogenesis in our model of IH. Both drugs acted in a dose-dependent manner without side effects related to antagonism on β2-adrenergic receptors, such as hypoglycemia or weight loss, in immunodeficient mice.

## Discussion

In this study, we demonstrate that R(+) enantiomers of the nonselective β-adrenergic receptor antagonist propranolol and the selective β1-adrenergic receptor antagonist atenolol inhibited HemSC vessel formation in vivo in a dose-dependent manner. Furthermore, we show that, like R(+) propranolol (23), R(+) atenolol inhibited HemSC to endothelial differentiation in vitro. R(+) propranolol did not inhibit HemSC to pericyte differentiation, indicating that the mechanism of action is specific to endothelial differentiation. Since the R(+) enantiomers of propranolol and atenolol lack beta blocker activity, these results indicate that propranolol and atenolol inhibited IH independently of β-adrenergic receptors.

Building on our previous findings (23), we now provide an in-depth molecular mode of action of R-enantiomer inhibition. Our study provides multiple lines of evidence that all converge toward the disruption of SOX18 transcriptional activity, including the use in vivo of a SOX18-specific small-molecule inhibitor (Sm4), which phenocopied the effects observed for both enantiomers. What we believe is novel is the demonstration that bypassing beta blockade using a molecular strategy that blocks SOX18 activity is powerful enough to halt IH progression in a preclinical model system. Further, we characterized an interference with protein partner recruitment (RBPJ) and also determined that the perturbation caused by R-propranolol was at the level of SOX18 population density in the chromatin-bound fraction. The use of SMT technology is at the cutting edge of what is currently available to assess transcription factor molecular behavior. The advantage of using real-time quantitative molecular imaging of the target protein is the establishment of a direct proof of on-target engagement by R-propranolol, hence leaving no doubt about the molecular mechanism at the genomic level.Our experiments were based on the premise that HemSCs isolated from proliferating-phase IH tissue are the cellular driver of hemangiogenesis. We purified HemSCs using antibodies against CD133, a cell-surface glycoprotein expressed on human stem and progenitor cells. In vitro, we found that HemSCs were capable of self-renewal and endothelial, pericytic, and adipogenic differentiation. In vivo, HemSCs formed glucose transporter 1^+^ (GLUT1^+^) blood vessels, a hallmark of IH, within 7 days and adipocytes within 28 days after injection into immunodeficient mice. Thus, HemSCs recapitulate key features of IH (27) and have been shown to be a target of corticosteroid (33), a prior therapy for IH (9, 44). Several laboratories have isolated HemSCs and confirmed their IH-forming capability (34, 45, 46).

The nonselective β-adrenergic receptor blocker propranolol is the current first-line therapy and only FDA-approved drug for treatment of IHs; however, its mechanism of action is not fully understood. Several in vitro studies provide clues. For example, propranolol inhibits HemSC proliferation in a dose-dependent manner and accelerates adipogenesis (47). This was confirmed by Li et al., who showed that propranolol promotes the differentiation of HemSCs into adipocytes (48). Propranolol (10 μM) did not inhibit HemSC proliferation (30, 36). The effect of propranolol on differentiated HemECs has been investigated in several studies. High concentrations (100 μM) of propranolol reduced the proliferation of HemECs by arresting cell progression at the G_0_/G_1_ phase and inducing apoptosis via the Akt pathway (49–51) and via mitochondrial stress (52). In our studies, we found a small antiproliferative effect of propranolol (10 μM) on HemECs (36). Thus, the potency of the antiangiogenic effect of propranolol on HemECs is not fully understood. HemPericytes display proangiogenic properties, high levels of VEGF-A, and a decreased ability to stabilize endothelium because of reduced angiopoietin 1 levels (35). Propranolol was shown to increase the contractility of HemPericytes in vitro (36), but, to date, the impact of propranolol on the differentiation of HemSCs into HemPericytes has not been investigated. Our murine model of IH showed comparable vessel formation and pericyte coverage when HemSCs and HemECs were coinjected into nude mice and treated with R(+) propranolol or PBS. Thus, it appears that R(+) propranolol does not affect pericyte coverage in vivo. Furthermore, propranolol and its enantiomers did not affect the differentiation of HemSCs into pericytes in vitro.

SOX18 is a well-studied transcription factor that controls vascular growth in development and disease (37). SOX18 homodimers and heterodimers regulate endothelial transcription (42) and play fundamental roles in arterial specification (53), lymphangiogenesis (24), and tumor angiogenesis (26). The disruption of SOX18 protein-protein interactions by small molecules is known to modulate its transcriptional activity, in particular its interference with the SOX18 homodimer by Sm4 (38). The homodimerization process of SOX18 is mediated via a 50-amino-acid-long DIM domain directly adjacent to the C-terminus of the high mobility group box (HMG-box, DNA binding region; ref. 42). Interestingly other PPIs such as RBPJ are mediated via the third α helix of the HMG-box (37); this suggests that the mode of action at the protein interface with R(+)-enantiomers is likely to differ between homodimers and heterodimers and might account for differential biological effects.

We demonstrated that propranolol can disrupt SOX18 protein partner recruitment and its transcriptional activity (23), which revealed an additional target for propranolol. In the same study, we showed that propranolol and its R(+) enantiomer inhibit HemSC to endothelial differentiation in vitro (23). Supporting the beta blocker–independent mechanism, R(+) propranolol rescued the neovascular defect in the SOX18 dominant-negative mutant Ragged Opossum mouse (23). Here, we extended these findings to in vivo HemSC vasculogenesis. HemSCs implanted into immunodeficient mice and treated with R(+) propranolol or R(+) atenolol exhibited a substantial reduction of vessel formation in a dose-dependent manner, establishing a β-adrenergic receptor–independent mechanism of action. Additionally, Sasaki et al. identified R(+) propranolol effects using middle T-antigen–transformed murine ECs derived from endothelioma (bEnd.3 cells; ref. 54). It was recently reported that propranolol ameliorates cavernous malformations in zebrafish by β1-adrenergic antagonism (55). Further, a central role for Sox18 during zebrafish arteriovenous specification (56, 57) and angiogenesis (25) has been reported, and it is therefore possible that in this model organism, a Sox18-dependent mechanism mediated via propranolol was also at play. Oral propranolol reduced lesion burdens in murine models of cerebral cavernous malformation 3, however the effect of the propranolol enantiomers was not investigated in the murine model (55).

Propranolol is a nonselective antagonist of β-adrenergic receptors that binds with high affinity to both β1- and β2-adrenergic receptor subtypes. Ji et al. (58) reported that, because of the effect of propranolol on β-adrenergic receptors, 2.1% of pediatric patients experienced intolerable side effects, resulting in the discontinuation of propranolol treatment. Side effects comprised sleep disorders (65.4%), severe respiratory disorders (15.4%), agitation (11.5%), and hypoglycemia (7.7%; ref. 58). Propranolol is a lipophilic beta blocker and is thus able to cross the blood-brain barrier, which may explain the sleep disorder and possibly neurodevelopmental or cognitive side effects (59). Notably, it has been shown that the R(+) enantiomer of propranolol crosses the blood-brain barrier to a lesser degree than propranolol or its S(–) enantiomer (60).

Atenolol, a hydrophilic selective beta-1 blocker, is used clinically to reduce potential side effects, because it has a limited ability to cross the blood-brain barrier and does not act on pulmonary or pancreatic β2-adrenergic receptors (16). Therefore, atenolol and its enantiomers do not interfere with the regulation of gluconeogenesis, glycogenolysis, or lipolysis. In our murine IH model, 12.5 mg/kg doses of the R(+) enantiomers of propranolol and atenolol did not affect the body weight or glucose levels of immunodeficient mice. Several studies confirmed that atenolol is safe and effective, yet side effects related to the blocking of β1-adrenergic receptors, e.g., hypotension and bradycardia, are still a concern, as in propranolol therapy (16, 61–63). Compared with propranolol (Hemangeol), there are few studies on the long-term effects and mechanism of action of atenolol. Like propranolol, atenolol exists in a racemic mixture of R(+) and S(–) enantiomers, with the R(+) enantiomer being mostly devoid of beta blocker activity (21). Our findings suggest that propranolol and atenolol converge toward a similar mechanism of action that does not rely on beta blocker activity. In our study R(+) atenolol inhibited HemSC to HemEC differentiation in vitro, disrupted SOX18 protein partner recruitment, and prevented IH vessel formation in vivo. R(+) atenolol was given at the same concentration as R(+) propranolol, suggesting that either enantiomer would be effective in the treatment of IH. This observation is crucial, because we show here that propranolol had direct effects inside the cell nucleus to alter a key regulator of endothelial genome activity. Since propranolol has been deemed safe in children, this suggests that long-term exposure of the healthy vasculature to a SOX18 inhibitor may not yield major adverse events.

Propranolol is administered at a dose of 2–3 mg/kg/day to pediatric patients, whereas atenolol is given at 1–2 mg/kg/day (5, 16, 62). In this study the lower dose of 2 × 5 mg/kg/day propranolol was chosen on the basis of a previous study, in which we showed that propranolol reduced vascular volume in implants containing HemECs and HemPericytes but had no effect on the number of vessels formed (36). The maximum dose of 2 × 12.5 mg/kg/day in mice is the animal-equivalent dose of 2 mg/kg/day propranolol in humans (32) and has shown an almost complete rescue of the vascular phenotype in a murine model of hypotrichosis-lymphedema-telangiectasia syndrome and renal syndrome caused by a SOX18 mutation (25 mg/kg/day was given once a day in the Ragged Opossum mouse model) (23).

There are several limitations to our study. An important one is that the murine IH model may not capture all aspects of hemangiogenesis, but it does recapitulate the formation of GLUT1^+^ vessels followed by adipogenesis, both hallmarks of IH. At present, it is the closest animal model for human IH vessel formation currently available. We did not carry out long-term studies to determine whether adipogenesis in the implants was accelerated by propranolol treatment, as has been proposed in some studies. The effect of the R(+) enantiomers of propranolol and atenolol on adipogenesis in the IH murine model could be addressed in future studies. We did not test the S(–) enantiomers of propranolol and atenolol for effects on HemSC-driven vessel formation in vivo, because these enantiomers would exert potent anti–β-adrenergic receptor and anti-SOX18 effects; this would reduce our ability to interpret the results. Another limiting factor is that HeLa cells were used in the Halo-SOX18 studies instead of HemSCs or HemECs. Unlike ECs, HeLa cells express very low levels of SOX18, which means there is little endogenous dark SOX18 to compete with the Halo-tagged SOX18. Thus, HeLa cells provide an advantageous setting for SOX18 SMT combined with pharmacological approaches.

Despite these limitations, our study suggests that, since R(+) enantiomers had a limited effect on β-adrenergic receptors, patients could be given a higher dose of R(+) propranolol or R(+) atenolol without increasing the risk of side effects. The metabolic data on mice in our study support this notion. Thus, the overall response rate of nearly 60% achieved with propranolol therapy in patients with IH might be significantly increased without an increase in side effects.

In conclusion, the R(+) enantiomers of propranolol and atenolol disrupted SOX18 transcriptional activity and inhibited the ability of HemSCs to undergo endothelial differentiation in vitro and vasculogenesis in vivo. We propose that the mechanism of drug action for both propranolol and atenolol, when used to treat IH, includes SOX18 and does not require β-adrenergic receptors. Use of the R(+) enantiomers could increase safety and efficiency by reducing β1- and β2-related side effects in the treatment of IH and possibly other types of vascular anomalies in which SOX18 plays a role (64, 65).

## Methods

### Cell isolation and culturing.

The clinical diagnosis of IH was confirmed in the Department of Pathology of Boston Children’s Hospital. Single-cell suspensions prepared from different proliferating-phase IH specimens were deidentified and designated as 150A, 165, or 167. HemSCs or HemECs were selected using anti-CD133– and anti-CD31–coated magnetic beads (Miltenyi Biotec), respectively, and expanded as described previously (27, 66). Testing for mycoplasma contamination by qPCR was performed when cells were thawed and every 4–6 weeks thereafter. Cells were cultured on fibronectin-coated (0.1 μg/cm^2^; MilliporeSigma) plates with Endothelial Growth Medium-2 (EGM-2; Lonza), which consists of Endothelial Cell Growth Basal Medium-2 (EBM-2; Lonza), SingleQuot supplements (all except hydrocortisone; Lonza), 10% heat-inactivated FBS (Hyclone), and 1× GPS (292 mg/mL glutamine, 100 U/mL penicillin, 100 mg/mL streptomycin; Mediatech). Cells were cultured at 37°C in a humidified incubator with 5% CO_2_.

### In vivo murine model for human blood vessel formation.

A stock solution of propranolol (169 mM; MilliporeSigma), R(+) propranolol (169 mM; MilliporeSigma), atenolol (3.75 mM; MilliporeSigma), and R(+) atenolol (7.51 mM; MilliporeSigma) was prepared in ddH_2_0. The stock solution was diluted with PBS to the indicated treatment concentrations. Experiments were carried out with 3 × 10^6^ HemSCs per implant, as described previously (27, 29). In vivo, the HemSCs undergo vasculogenesis and form anastomoses with ingrowing host vessels; the implants do not expand in size. HemSCs (150A) were grown in EGM-2 medium until subconfluent. Twenty-four hours before harvesting, 10 μM treatment drug or PBS as a control was added to the media. Cells were counted after the 24-hour pretreatment and suspended in 200 μL Matrigel (Corning) preadjusted with 1 μg/mL basic FGF (bFGF) (ProSpec), 1 μg/mL erythropoietin (EPO) (ProSpec), and 5 μM drug or PBS on ice. The Matrigel/cell suspensions were injected s.c. into the backs of 6-week-old male athymic nu/nu mice (Massachusetts General Hospital, Boston, Massachusetts, USA), placing 2 implants per mouse (*n =* 3–5 mice/group; see schematic Figure 1A). The mice were given 5 mg/kg or 12.5 mg/kg propranolol, atenolol, their respective R(+) enantiomers, or PBS as a control (200 μL/mouse, i.p.) twice a day. Sm4 (SML1999; MilliporeSigma) was administered by oral gavage at a concentration of 25 mg/kg once a day. For the control, 10% DMSO in PBS was used. Body weight and blood glucose levels of the mice were measured daily before the morning i.p. injection or oral gavage for the duration of each respective experiment. Glucose concentrations were measured in tail vein blood using the OneTouch UltraSmart Blood Glucose Monitoring System (LifeScan). After 7 days, the mice were euthanized and the implants were removed, fixed in formalin, embedded in paraffin, and analyzed by H&E staining and IF. Blood vessels (indicated by the luminal structures containing 1 or more red blood cells) and CD31^+^ human vessels were counted in 5 fields/section, 2 sections/implant. Each field was 425.1 μm × 425.1 μm = 0.18071 mm^2^, and sections were from the middle of the implant. Vessel density is expressed as vessels/mm^2^. For perivascular cell coverage, the vessel area was measured with Fiji ImageJ software (NIH), and surrounding mural cells were counted in 5 fields/section, 2 sections/implant.

### Histology and IF analysis.

FFPE tissue sections (5 μm thick) of the Matrigel implants were deparaffinized and either directly stained with H&E or immersed in an antigen retrieval solution (citrate-EDTA buffer containing 10 mM citric acid, 2 mM EDTA, and 0.05% Tween-20, pH 6.2) for 20 minutes at 95°C–99°C. Sections were subsequently blocked for 30 minutes in TNB Blocking Buffer (PerkinElmer) followed by incubation with human-specific CD31 monoclonal antibody (1:30, mouse anti-human; Dako, Glostrup, 0823) to stain human ECs, anti–αSMA (1:1000, mouse anti-human, MilliporeSigma, A5228) to stain perivascular cells or the plant lectin *Ulex europaeus* agglutinin 1 (UEA1) to stain human ECs (1:50; Vector Laboratories, FL-1061) (67). UEA1 was fluorescently labeled with FITC. Next, sections were incubated with Alexa Fluor 546 donkey anti–mouse IgG (1:200, Invitrogen, Thermo Fisher Scientific, A10036) as a secondary antibody.

FFPE tissue sections (5 μm) from patients with IH were deparaffinized, immersed in an antigen retrieval solution, and blocked for 30 minutes followed by incubation with anti-SOX18 (1:50; Santa Cruz Biotechnology, sc-376166), anti-RBPJ fluorescently labeled with Alexa Fluor 546 (1:50; Santa Cruz Biotechnology, sc-271128 AF546), and UEA1 fluorescently labeled with Alexa Fluor 649 **(**1:50; Vector Laboratories, FL-1061). Next, the sections were incubated with Alexa Fluor 488 donkey anti–mouse IgG (1:200; Invitrogen, Thermo Fisher Scientific, A10036) as a secondary antibody. All slides were mounted using DAPI (Molecular Probes, R37606) to visualize nuclei.

IF Images were acquired by a Zeiss Airyscan LSM 880 Fast confocal microscope, and H&E images were analyzed through a ×20 objective lens using a Zeiss Axio Imager M1 microscope (Carl Zeiss Microscopy). All images were analyzed using Fiji ImageJ software.

### HemPericyte differentiation assay.

The in vitro pericytic differentiation assay was performed by seeding HemSCs (150A) together with HemECs (150A) at a 1:1 ratio and a total density of 3 × 10^4^ cells/cm^2^ on fibronectin-coated plates in EGM-2, as previously described (28). PBS (Lonza) was added to the medium of the negative control and 25 μM DAPT (MilliporeSigma) to the medium as a positive control for inhibition. A stock solution of 50 mg propranolol (MilliporeSigma) or R(+) propranolol (MilliporeSigma) in 1 mL ddH_2_0 (pH 3.0; 169 mM) was prepared. Ten micromolars propranolol or R(+) propranolol was added to the media, and the media were replaced every other day. The cells were cocultured with the added drug for 5 days. After coculturing, HemECs were removed from the trypsinized cell suspension with anti-CD31–coated dynabeads (Invitrogen, Thermo Fisher Scientific) according to the manufacturer’s depletion protocol (see schematic in Figure 2C), and the resulting CD31^–^ cells were used for further analysis.

### Hemangioma endothelial differentiation assay.

To induce endothelial differentiation, HemSCs (types 165 and 167) were seeded on fibronectin-coated plates at a density of 20,000 cells/cm^2^ in EGM-2. After 18–24 hours, the medium was replaced with serum-free EBM-2 containing 10 ng/mL VEGF-B (R&D Systems), 1× insulin transferrin-selenium, 1:5000 linoleic acid–albumin, 1 mM dexamethasone, and 60 mM ascorbic acid–2–phosphate (68). A stock solution of 10 mM propranolol hydrochloride (MilliporeSigma), R(+) propranolol hydrochloride (MilliporeSigma), atenolol (MilliporeSigma), and R(+) atenolol (MilliporeSigma) was prepared in DMSO (MilliporeSigma). Cells were cultured in the VEGF-B, serum-free media, with or without the indicated drugs, at a concentration of 5 μM propranolol, atenolol, and their R(+) enantiomers for 8 days. DMSO without VEGF-B served as a negative control and DMSO with VEGF-B as a positive control.

### RNA isolation and qPCR.

Total cellular RNA was extracted from cells with an RNeasy Micro Extraction Kit (QIAGEN). Reverse transcriptase reactions were performed using an iScript cDNA Synthesis Kit (Bio-Rad). qPCR was performed using Kapa SYBR FAST ABI Prism 2× qPCR Master Mix (Kapa BioSystems). Amplification was carried out in a StepOne Real-Time PCR System (Applied Biosystems). A relative standard curve for each gene amplification was generated to determine the amplification efficiency, with greater than 90% considered acceptable. Fold increases in gene expression were calculated according to the ΔΔCt method, with each amplification reaction performed in duplicate or triplicate (69, 70). Gene expression was normalized to the PBS treatment. *ATP5B* was used as housekeeping gene expression reference. Primer sequences are shown in Table 1.

### VCAM1 promoter fragment luciferase reporter assay.

COS-7cells (7000 cells/well) were seeded in gelatin-coated 96-well plates (Gibco DMEM, Thermo Fisher Scientific, 10% v/v heat-inactivated FBS, 1% l-glutamine, penicillin, and streptomycin). Cells were maintained at 37°C in 5% CO_2_. After 24 hours, a 4-hour transfection with murine plasmids, a pGL2-Basic (Promega) Vcam-1 promoter construct (VC1889), and pSG5 SOX18 was performed (40 ng of each plasmid per well in 10 μL Premix X-tremeGENE HP DNA Transfection Reagent, Roche/MilliporeSigma, 1:4 DNA/X-tremeGENE ratio; ref. 39). Drugs were added at noncytotoxic concentrations (0.3% DMSO v/v), and cells were incubated for another 18 hours in low-serum media (0.5% v/v), followed by cell lysis and measurement of luciferase activity with a PerkinElmer Neolite Assay Kit. Three independent experiments were performed, and compounds were tested in technical quadruplicates on each occasion. The compounds’ apparent EC_50_ were estimated using GraphPad Prism 3-parameter nonlinear regression analysis (GraphPad Software, version 8.4.3).

### AlphaLISAScreen technology.

Proteins were genetically encoded with N-terminal enhanced GFP or C-terminal mCherry tags and cloned into cell-free expression gateway destination vectors (71). Human ORFs were sourced from various human ORFeome collections (Harvard version 1.1, version 5.1, and OCAA), and cloned by Gene Universal Inc. or Genscript Inc. Translation-competent *Leishmania tarentolae* extract (LTE) was purchased from Jena Bioscience GmbH and prepared as previously described by Kovtun et al. (72). An AlphaLISAScreen was performed as previously described (23). Cell-free expression was analyzed on OptiPlate-384 plates (PerkinElmer). AlphaLISA coupling was performed in ProxyPlate-384 plates (PerkinElmer), as described by the manufacturer, and involved robotically assisted stepwise addition of anti-GFP AlphaLISA acceptor beads, a biotinylated anti-mCherry nanobody, and streptavidin donor beads (PerkinElmer). The plates were incubated for 90 minutes at room temperature before AlphaLISA signal was measured on a TECAN Spark plate reader (AlphaLISA mode, 130 ms excitation, 300 ms integration). Each protein pair was measured at 2 dilutions in triplicate on 3 independent occasions. Only 1 dilution was selected for data analysis. A DMSO vehicle control (0% inhibition) and a SOX18:FRB control (no known protein interaction, i.e., 100% inhibition) were analyzed in technical quintuplets on each occasion to normalize the inhibition data.

### SMT assay.

SMT was performed as described in McCann et al. (41). pReceiver-M49 (HaloTag-SOX18) was obtained from GeneCopoeia. HeLa cells were a gift from Geoffrey Faulkner (Queensland Brain Institute/Translational Research Institute, St. Lucia, Queensland, Australia). Cells were cultured in DMEM (Gibco, Thermo Fisher Scientific) supplemented with 10% FBS (GE Healthcare), 1% GlutaMAX (Gibco, Thermo Fisher Scientific), and 1% MEM Non-Essential Amino Acids (MEM NEAA, Gibco, Thermo Fisher Scientific). Cells were maintained at 37°C with 5% CO_2_. HeLa cells were seeded at a density of 20,000 cells/well in 8-well chamber glass slides (Ibidi) coated with 0.5% gelatin 24 hours prior to transfection. Transfections were performed using the X-tremeGENE 9 Transfection Reagent Kit (Roche) to introduce 300 ng plasmid DNA according to the manufacturer’s instructions, using FluoroBrite DMEM (Gibco, Thermo Fisher Scientific) supplemented with 1% GlutaMAX (Gibco, Thermo Fisher Scientific) as the low-serum transfection media. Cells were incubated at 37°in 5% CO_2_ for 24 hours prior to imaging. Three hours before imaging, cells were treated with either 20 μM R(+)propranolol dissolved in PBS or an equivalent volume of PBS alone. Immediately prior to imaging, cells were washed twice and replaced with FluoroBrite DMEM (Gibco, Thermo Fisher Scientific) imaging media. JF549 HaloTag dye (2 nM) was subsequently added directly to the media, and cells were incubated for 10 minutes at 37°C in 5% CO_2_. Following incubation, cells were washed twice and replaced with FluoroBrite DMEM (Gibco, Thermo Fisher Scientific). Images were acquired on a Nikon TIRF microscope at a TIRF angle of 60.18 degrees to achieve HILO illumination. Samples were recorded with an iXon Ultra 888 EMCCD camera, a filter cube TRF49909 – ET – 561 laser bandpass filter, and ×100 oil 1.49 NA TIRF objective. Cells were imaged using a 561 nm excitation laser at a power density of 10.3 μW to perform 2 different acquisition techniques. A fast frame rate, which uses 50 Hz (20 ms acquisition speed) to acquire 6000 frames without intervals, was applied to measure displacement distribution and bound fractions, and a slow frame rate, which uses a 2 Hz (500 ms acquisition speed) to acquire 500 frames without intervals, was applied to measure residence times. All images were cropped, and nuclei areas were measured using ImageJ (fast and slow tracking). Molecules were identified using a custom-written MATLAB implementation of the MTT algorithm (73), known as SLIMfast (74). The parameters used for fast frame rate analysis were as follows: localization error, 10^–6.5^; blinking (frames), 1; maximum number of competitors, 3; and maximum expected diffusion coefficient (μm^2^/s), 2. SpotOn, a model-based analysis of single-particle tracking, was used to determine diffusion coefficients and population fractions for the 2-state kinetic model (75). The parameters used for slow frame rate analysis were as follows: localization error, 10^–7^; blinking (frames): 1; maximum number of competitors, 3; and maximum expected diffusion coefficient (μm^2^/s), 0.1. Slow-tracking analysis was performed using MATLAB code written by Chen and colleagues (40). The number of trajectories per nucleus is an output of the custom-written script from Chen et al. The density of trajectories per nucleus was calculated by dividing the number of trajectories by the area of the nucleus associated with an individual sample.

### Statistics.

Data were analyzed and plotted using GraphPad Prism 9.1 (GraphPad Software). Results are displayed as the mean ± SD unless otherwise indicated. For experiments in which cells were treated with alternative drugs, the differences were assessed by 1-way ANOVA followed by Bonferroni’s or Tukey’s post hoc test for multiple comparisons of different treatment modalities or Dunnett’s test for multiple comparisons to compare every treatment mean with that of the PBS control. For comparisons between treatment and control groups, 2-tailed, unpaired Student *t* tests were applied. For SMT experiments, the results are displayed as individual values with the mean ± SD or as the frequency distribution. Significance was assessed by either Welch’s *t* test or a 2-way ANOVA with Šidák multiple-comparison correction.

### Study approval.

Animal protocols complied with NIH Animal Research Advisory Committee guidelines and were approved by the Boston Children’s Hospital Animal Care and Use Committee (protocol number 19-09-4008R). IH specimens were obtained under a protocol approved by the Committee on Clinical Investigation at Boston Children’s Hospital (IRB protocol number 04-12-175R). Hemangioma specimens were collected upon written informed consent of the guardian, deidentified, and used for cell isolation under a Boston Children’s Hospital IRB approved protocol (04-12-175R) and in accordance with Declaration of Helsinki principles.

## Author contributions

CTS, LH, MF, and JB designed the study. CTS, AM, JWS, LH, FF, and TK performed the experiments. CTS, MSG, AM, LH, JWS, DZ, SJS, JB, and MF analyzed the data. FM contributed to quality control of SMT data sets and software used for analysis. CTS, FF, MF, and JB wrote the manuscript. LH, DZ, JBM, MF, and JB revised and edited the manuscript. All authors read and agreed to the final version of the manuscript.

## Supplementary Material

Supplemental data

## Figures and Tables

**Table 1:**
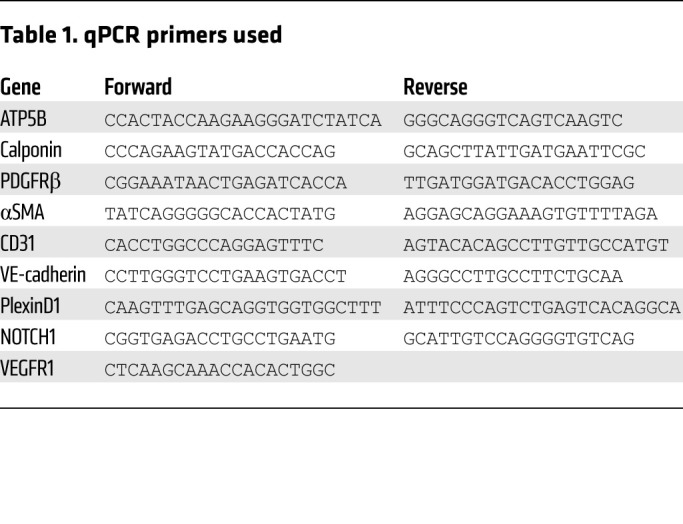
qPCR primers used

## Version 1 12/07/2021

In-Press Preview

## Version 2 02/01/2022

Electronic publication
