# Supplementary material for: Non–beta blocker enantiomers of propranolol and atenolol inhibit vasculogenesis in infantile hemangioma
Source: J Clin Invest. 2022 Feb 1;132(3):e151109. doi: 10.1172/JCI151109 (PMC8803322; doi:10.1172/JCI151109)
Supplement: Supplemental data [file jci-132-151109-s088.pdf]

Non-β-Blocker Enantiomers of Propranolol and Atenolol Inhibit Vasculogenesis in Infantile Hemangioma

Caroline T. Seebauer[1], Matthew S. Graus[2]*, Lan Huang[1]*, Alex McCann[3]*, Jill Wylie Sears[1], Frank Fontaine[4], Tara Karnezis[4], David Zurakowski[5], Steven J. Staffa[5], Frederic Meunier[3], John B. Mulliken[1], Joyce Bischoff[1]** and Mathias Francois[2]**

10. Supplemental tables and figures

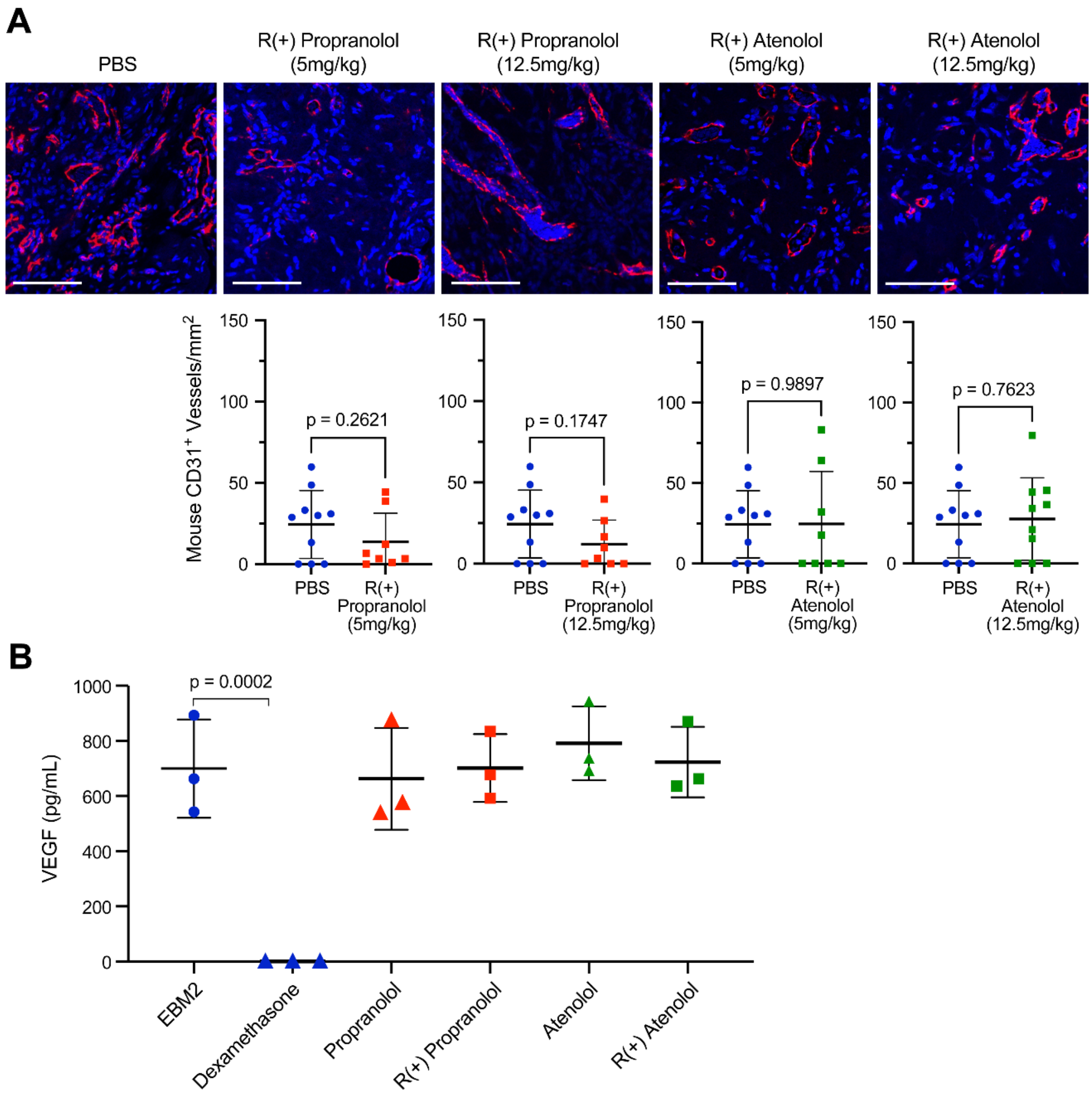

Supplemental Figure 1. R(+) enantiomers do not affect host (mouse) vessels in a murine model for IH. (A) HemSC were pretreated with PBS or 10uM R(+) propranolol for

24h, suspended in Matrigel with PBS or 5uM R(+) propranolol and injected into nude mice, two implants/mouse (N=8). The mice were treated with 5mg/kg R(+) propranolol, 12.5mg/kg R(+) propranolol, 5mg/kg (R+) atenolol, 12.5mg/kg R+) atenolol or an equivalent volume of PBS twice a day. Matrigel implants were harvested after 7 days. Anti-mouse CD31 (Clone MEC13.3, BD Biosciences Pharmingen) staining (red) confirmed similar vessel density of host (murine) vessels in treated mice compared to control mice. Nuclei counterstained with DAPI (blue). Scale bar, 100µm. P-values were calculated by two-tailed, unpaired student-t test. Means and standard deviations are shown. (B) HemSC (150A) were treated for three days with medium alone (negative control), 2uM dexamethasone (positive control), 5uM propranolol, R(+) propranolol, atenolol, and R(+) atenolol . VEGF-A protein levels in the conditioned media were quantified using the Quantikine Human VEGF kit (R&D Systems enzyme-linked immunosorbent assay) as previously described (33). Neither propranolol, atenolol, nor their R(+)enantiomers affected VEGF-A secretion of HemSC.

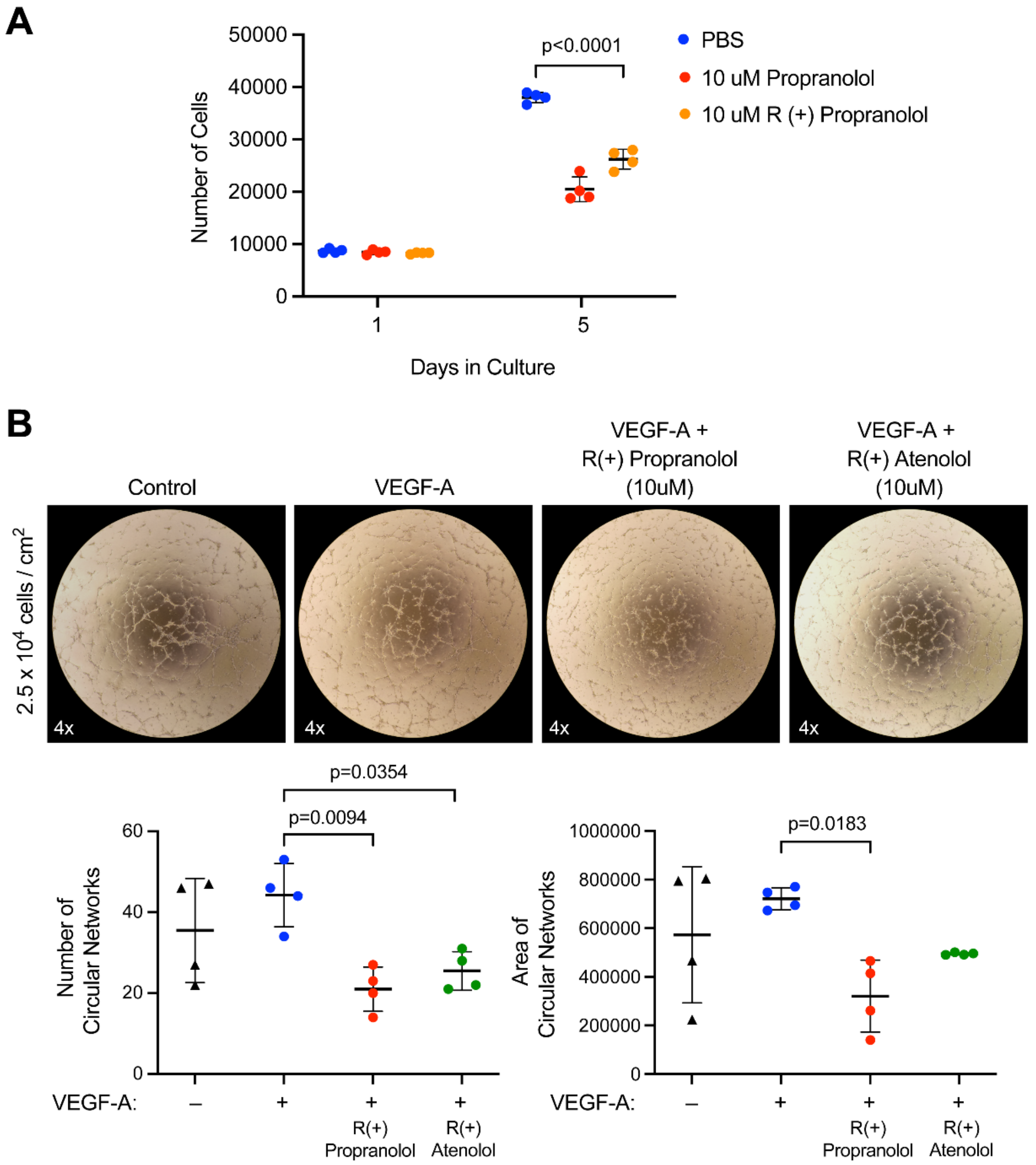

C

Supplemental Table for Figure 2C (p-values) One-way ANOVA with Dunnett's multiple comparisons test			
	Calponin	αSMA	PDGFRβ
PBS vs DAPT	p = 0.0139	p = 0.3932	p = 0.0063
PBS vs Propranolol	p = 0.4575	p = 0.8137	p = 0.9998
PBS vs R(+) Propranolol	p = 0.5776	p = 0.9912	p = 0.1746

Supplemental Figure 2. Effect of R(+) propranolol on tube formation and proliferation of HemEC. (A) HemEC (150A) were plated at 5000 cells/cm^2 in 48-well plates in EGM-2 (n = 4/condition). Cells were treated twice daily with PBS, 10uM propranolol, R(+) propranolol or S(-) propranolol from days 1 to 5. Cells were counted using an automated cell counter

(Scepter 2.0; Millipore, Milford, MA, U.S.A.); all particles with diameters ranging from 10 to 25 μm were included. Statistical analysis was done by two-way ANOVA with Tukey's multiple comparisons test. (B) HemEC (150A) were pretreated with 10uM R(+) propranolol and R(+) atenolol (treatment groups), or equal amounts of DMSO (control groups), for one hour. 25ng/ml VEGF-A was added to one control group and the two treatment groups. Wells were precoated with Matrigel and incubated for 30 minutes at 37 °C. HemEC (150A) were seeded at a density of 2.5×10^4 cells/cm^2 in 500 μL of EBM-2/0.1%FBS. 25ng/ml VEGF-A and 10uM of respective treatment or equal amount of DMSO was added to the VEGF-A control and treatment conditions. After 6 hours, pictures were taken with 4x magnification with an inverted microscope (Echo Rebel Inverted Brightfield Microscope, Echo, San Diego, CA). The number of circular networks was counted per nine squares per high power field. The area of the circular networks was measured in pixels. Fiji ImageJ software (NIH) was used for analysis. Statistical analysis was done by one-way ANOVA with Tukey's multiple comparisons test. Means and standard deviations are shown in all graphs. (C) Supplemental Table for Figure 2C.

Supplemental Table for Figure 3A (p-values)<br>One-way ANOVA with Bonferroni's post-hoc test				
VEGF-B versus VEGF-B + R(+) propranolol				
CD31	CDH5	NOTCH1	VEGFR1	PLXND1
p < 0.0001	p < 0.0001	p = 0.0102	p = 0.093	p = 0.0009
VEGF-B versus VEGF-B + atenolol				
CD31	CDH5	NOTCH1	VEGFR1	PLXND1
p < 0.0001	p < 0.0001	p = 0.0104	p = 0.0434	p = 0.0004
VEGF-B versus VEGF-B + R(+) atenolol				
CD31	CDH5	NOTCH1	VEGFR1	PLXND1
p < 0.0001	p < 0.0001	p = 0.007	p = 0.0137	p = 0.0003

Supplemental Figure 3. (A) Supplemental Table for Figure 3A.

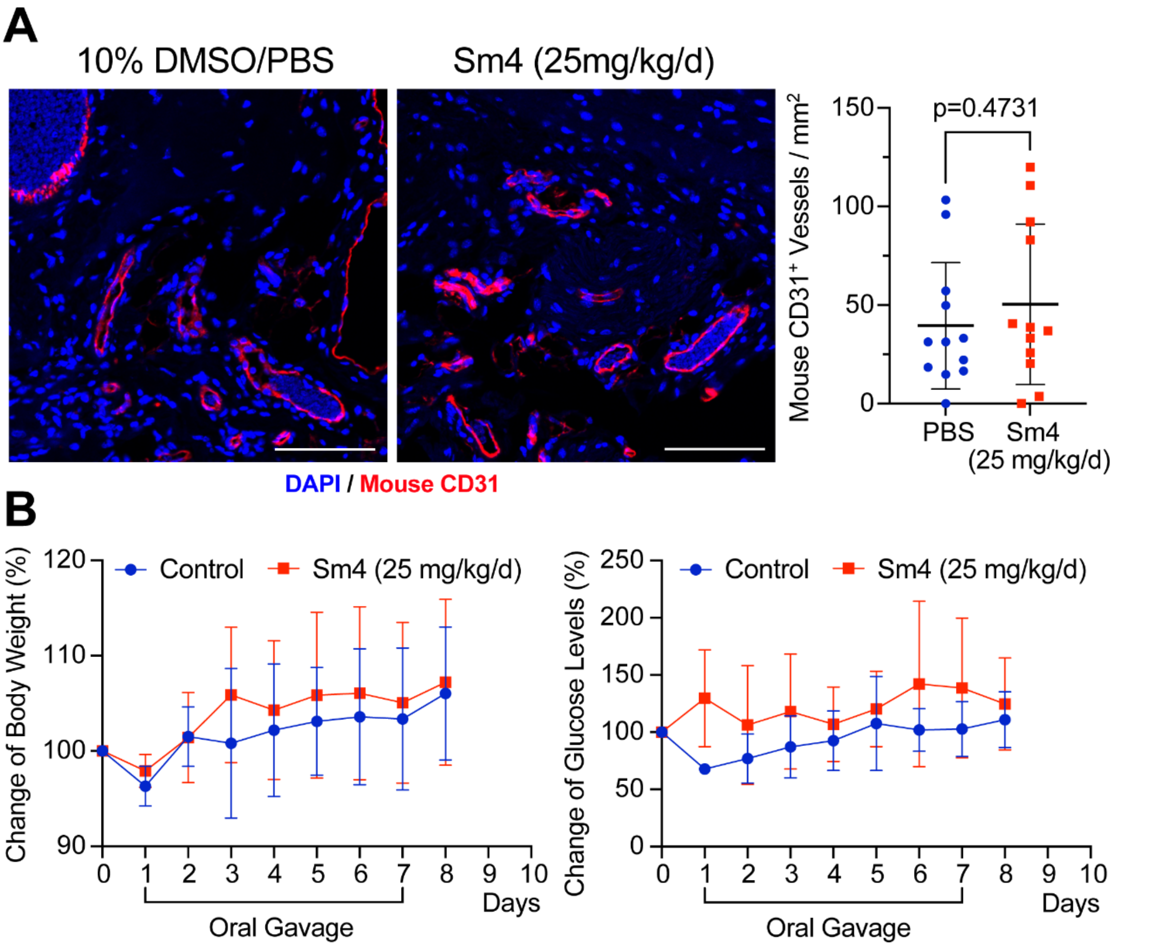

Supplemental Figure 4. Sm4 does not affect density of host (mouse) vessels in Matrigel implants or body weight or glucose levels. IH. (A) HemSC were pretreated with 10%DMSO/PBS or 10uM Sm4 for 24h, suspended in Matrigel with 10%DMSO/PBS or 5uM Sm4 and injected into nude mice, two implants/mouse. The mice were treated with 25mg/kg Sm4 once a day or an equivalent volume of 10%DMSO/PBS. Matrigel implants were harvested after 7 days. Anti-mouse CD31 (Clone MEC13.3, BD Biosciences Pharmingen) staining (red) confirmed similar vessel density of host (murine) vessels in Sm4-treated mice compared to control mice. Nuclei counterstained with DAPI (blue). Scale bar, 100µm. Data were collected for 2 implants in each of six mice, leading to a sample size of N=12 observations per group. P-values were calculated by two-tailed, unpaired student-t test. (B) Body weight and glucose levels were measured daily. Sm4 did not affect body weight or glucose levels of nude mice. Means and standard deviations are shown in all graphs.

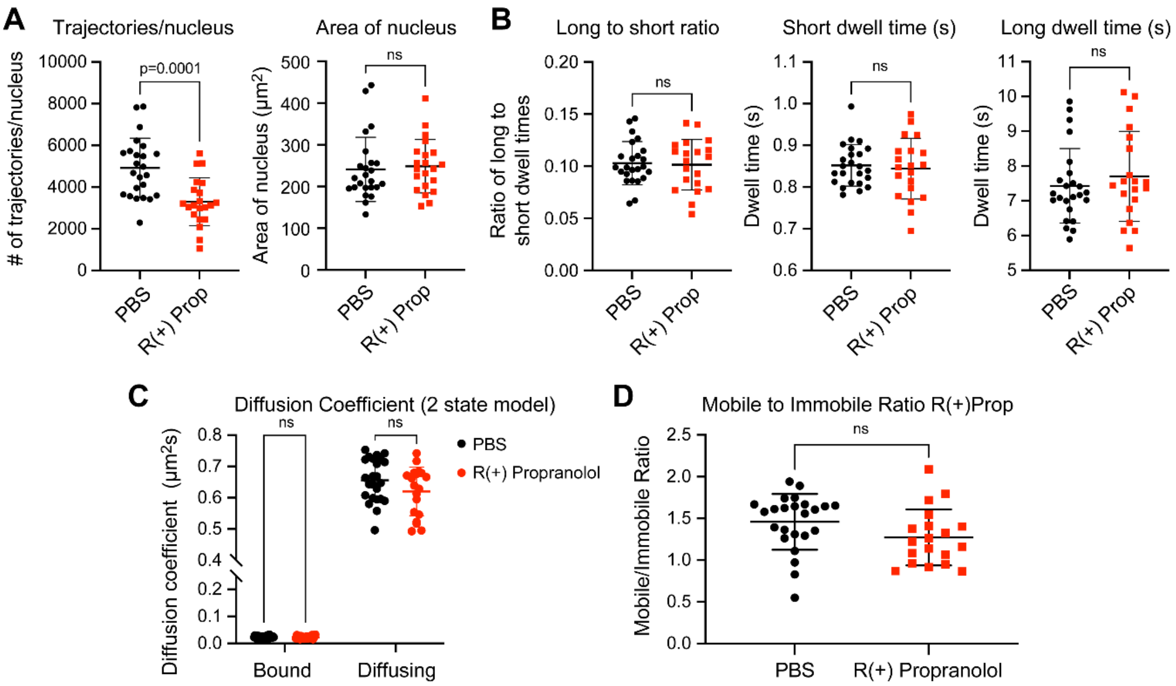

E

Supplemental Table for Figure 5B (p-values)<br>One-way ANOVA<br>with Bonferroni post-hoc test	
	VCAM1
DMSO vs VEGFB	p < 0.0001
VEGFB vs VEGFB/Sm4	p < 0.0001
VEGFB vs VEGFB/Propranolol	p < 0.0001
VEGFB vs VEGFB/R+ Propranolol	p < 0.0001

F

Supplemental Table for Figure 5D (p-values)<br>One-way ANOVA<br>with Dunnett's post-hoc test		
SOX18:RBPJ		
	p-values	% Inhibition
Control vs SOX18:FRB	p < 0.0001	100.00
Control vs Propranolol	p < 0.0001	42.70
Control vs Atenolol	p < 0.0001	45.46
Control vs R(+) Propranolol	p = 0.0002	29.18
Control vs R(+) Atenolol	p < 0.0001	36.88
SOX18:SOX18		
	p-values	% Inhibition
Control vs SOX18:FRB	p < 0.0001	100.00
Control vs Propranolol	p = 0.0016	29.47
Control vs Atenolol	p = 0.0077	25.47
Control vs R(+) Propranolol	p = 0.1312	16.52
Control vs R(+) Atenolol	p = 0.0533	19.71

Supplemental Figure 5. Single Molecule Tracking. HeLa cells transfected with Halo-tagged SOX18 were treated with PBS or R(+) propranolol and imaged as described in the methods. (A) Trajectories per nucleus and comparison of nucleus area. (B) Ratio of long to short dwell time, long dwell time, and short dwell time. (C) Diffusion coefficient of two-state

kinetic model and (D) the mobile to immobile ratio. Statistics for A and B, and mobile to immobile ratio (D) were determined by Welch's T-test. (C) Two-way ANOVA with Sidak multi-comparison correction was used to test significance for the diffusion coefficient (two-state model). based on four technical replicates with six cells per replicate per condition (n ≥ 20 cells). n.s indicates p > 0.05. (E) Supplemental table for Figure 5B. (F) Supplemental table for Figure 5D.

A

	No Treatment	Propranolol
Number of Patients	13	13
Proliferating Phase	4	4
Involuting Phase	5	5
Involuted Phase	4	4
Male	3	3
Female	10	10
Age at Surgery (median, in months)	31.1	28.9
Treatment Duration (median, in months)	n.a.	10.7
Age at First Dose (median, in months)	n.a.	2.5

B

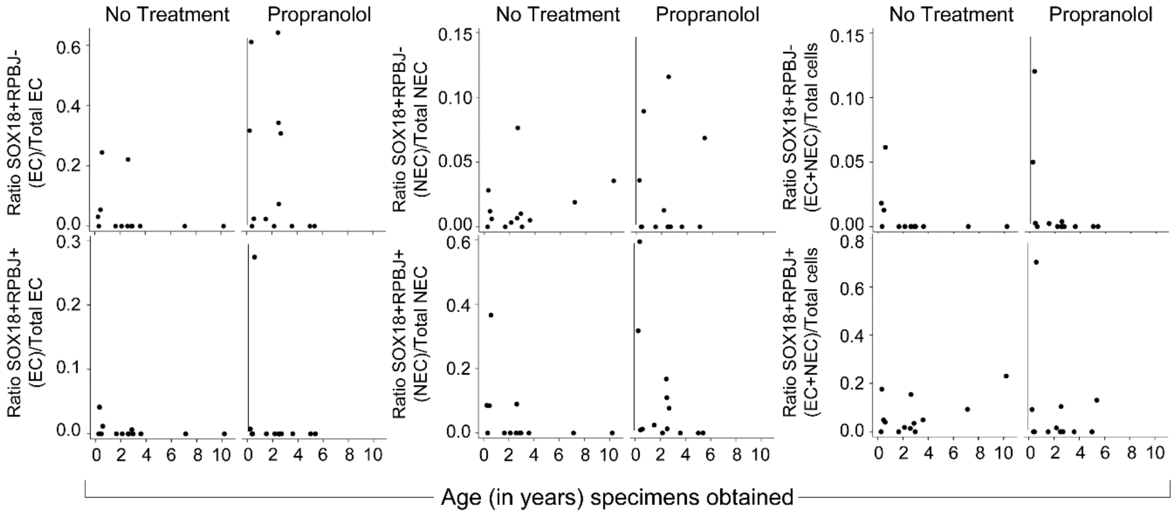

Supplemental Figure 6. Propranolol treatment does not affect number of SOX18 positive cells in human IH. Formalin-fixed, paraffin-embedded (FFPE) tissue sections (5µm) from IH were immunostained as in Figure 5F. (A) Patient characteristics of the cohort of 26 hemangioma patients with 13 age-matched pairs. 13 patients received propranolol treatment over a median duration of 10.7 months. 13 patients received no treatment. Medians of ages and duration are displayed in months. (n.a. = not applicable). (B) Quantification of endothelial cells positive for SOX18 (SOX18+RBPJ-), non-endothelial cells

(NEC) positive for SOX18 (SOX18+RBPJ-) and total cells positive for SOX18 (SOX18+RBPJ-) are shown in the top row. Endothelial cells positive for SOX18 and RBPJ, NEC positive for SOX18 and RBPJ and total cells positive for SOX18 and RBPJ are shown in the bottom row. No significant effect of propranolol was found on the number of SOX18+RBPJ- or SOX18+RBPJ+ cells per lesion. Quantile regression with clustering based on the matched pairs was used to determine p values.
